# Comparative chloroplast genome analysis of Impatiens species (Balsaminaceae) in the karst area of China: insights into genome evolution and phylogenomic implications

**DOI:** 10.1186/s12864-021-07807-8

**Published:** 2021-07-24

**Authors:** Chao Luo, Wulue Huang, Huayu Sun, Huseyin Yer, Xinyi Li, Yang Li, Bo Yan, Qiong Wang, Yonghui Wen, Meijuan Huang, Haiquan Huang

**Affiliations:** 1 College of Landscape Architecture and Horticulture Sciences, Southwest Research Center for Engineering Technology of Landscape Architecture(State Forestry and Grassland Administration), Yunnan Engineering Research Center for Functional Flower Resources and Industrialization, Research and Development Center of Landscape Plants and Horticulture Flowers, Southwest Forestry University, Kunming, Yunnan 650224 China; 2 Department of Landscape Architecture and Plant Science, University of Connecticut, Storrs, CT 06269 USA

**Keywords:** *Impatiens*, Balsaminaceae, Chloroplast genome, Comparative analysis, Phylogenetic relationship

## Abstract

**Background:**

*Impatiens* L. is a genus of complex taxonomy that belongs to the family Balsaminaceae (Ericales) and contains approximately 1000 species. The genus is well known for its economic, medicinal, ornamental, and horticultural value. However, knowledge about its germplasm identification, molecular phylogeny, and chloroplast genomics is limited, and taxonomic uncertainties still exist due to overlapping morphological features and insufficient genomic resources.

**Results:**

We sequenced the chloroplast genomes of six different species (*Impatiens chlorosepala, Impatiens fanjingshanica, Impatiens guizhouensis, Impatiens linearisepala, Impatiens loulanensis,* and *Impatiens stenosepala*) in the karst area of China and compared them with those of six previously published Balsaminaceae species. We contrasted genomic features and repeat sequences, assessed sequence divergence and constructed phylogenetic relationships. Except for those of *I. alpicola, I. pritzelii* and *I. glandulifera*, the complete chloroplast genomes ranging in size from 151,366 bp (*I. alpicola*) to 154,189 bp (*Hydrocera triflora*) encoded 115 distinct genes [81 protein-coding, 30 transfer RNA (tRNA), and 4 ribosomal RNA (rRNA) genes]. Moreover, the characteristics of the long repeat sequences and simple sequence repeats (SSRs) were determined. *psbK-psbI, trnT-GGU-psbD, rpl36-rps8, rpoB-trnC-GCA, trnK-UUU-rps16, trnQ-UUG, trnP-UGG-psaJ, trnT-UGU-trnL-UAA,* and *ycf4-cemA* were identified as divergence hotspot regions and thus might be suitable for species identification and phylogenetic studies. Additionally, the phylogenetic relationships based on Maximum likelihood (ML) and Bayesian inference (BI) of the whole chloroplast genomes showed that the chloroplast genome structure of *I. guizhouensis* represents the ancestral state of the Balsaminaceae family.

**Conclusion:**

Our study provided detailed information about nucleotide diversity hotspots and the types of repeats, which can be used to develop molecular markers applicable to Balsaminaceae species. We also reconstructed and analyzed the relationships of some *Impatiens* species and assessed their taxonomic statuses based on the complete chloroplast genomes. Together, the findings of the current study might provide valuable genomic resources for systematic evolution of the Balsaminaceae species.

**Supplementary Information:**

The online version contains supplementary material available at 10.1186/s12864-021-07807-8.

## Background

The nucleus, chloroplast (cp), and mitochondrion are the three major organelles containing genomes within the cell [1]. Typically, the chloroplast genomes in angiosperms display a quadripartite circular double-helix structure with highly conserved sizes, structures, and gene sequences ranging from 115 kb to 165 kb in length [2]. The complete chloroplast genome’s common feature is a typical tetrad structure consisting of a pair of inverted repeats (IRs) separated by the large and small single-copy regions (LSC and SSC regions, respectively). Generally, chloroplast genomes contain 110–113 genes, which are separated into three categories according to their functions [3]. The first is related to the expression of chloroplast genes such as transfer RNA (tRNA) genes, ribosomal RNA (rRNA) genes, and the three subunits associated with RNA polymerase synthesis. The second corresponds to photosynthesis-related genes, and the third to other biosynthetic genes and some genes of unknown function, such as *ycf1, ycf2* and *ycf15* [4]. Compared to the nuclear and mitochondrial genomes, the chloroplast genome has a self-replication mechanism, relatively independent evolution, a small genome, low mutation rate and unique maternal inheritance [5]. Thus, the chloroplast genome can provide information for the evolutionary analysis, DNA barcoding, phylogenetic reconstruction and taxonomic identification of families and generas [6]. Furthermore, gene mutations, rearrangements, duplications and losses could be observed in the chloroplast genomes of angiosperm lineages [7]. Structural changes in genomes can be used to study taxonomic significance and phylogenetic relationships [8] and can supply information for developing genomic markers for complex, taxonomically challenging species [9]. Complete chloroplast genomes contain all genes for the reconstruction of evolutionary history and can provide more valuable and higher-quality information for evolutionary and phylogenetic analyses [10]. In addition, they can also reduce the sampling error inherent in studies of one or a few genes that may indicate critical evolutionary events [11].

*Impatiens* species, belonging to Balsaminaceae, form a taxonomically controversial and complex genus of flowering plants that have been widely used as medicinal, ornamental, and horticultural plants in North America, Europe, and China [12]. Family Balsaminaceae consists of only two genera, namely, *Impatiens* and the monospecific sister genus *Hydrocera* (consisting of *Hydrocera triflora*; GenBank KF986530), with strong similarities in morphology and molecular biology [13]. Both are eudicot genera that belong to order Ericales and subclass Asteridae. The new classification of *Impatiens* based on morphological and molecular datasets divided it into two subgenera (*Clavicarpa* and *Impatiens*). Seven sections of the subgenus were further subdivided. *Impatiens* includes approximately 1000 species distributed from the tropics to the subtropics and extending from sea level to an altitude of 4000 m [14]. Tropical Africa, Madagascar, Sri Lanka, Himalayas, and Southeast Asia are the five biodiversity hotspots of *Impatiens* [15, 16].

The center of origin and diversification of Balsaminaceae is China, especially the karst area. Approximately 250 wild *Impatiens* species have been described from the Guizhou, Yunnan, and Guangxi areas, many of which are used as supplements for medicinal or health purposes. In ancient China, *Impatiens* plants were called ‘zhijiahua’ and were crushed into a mash and directly applied to the nails [17]. Pharmaceutical and chemical products of annual herbs can be used for the medical treatment of rheumatism, beriberi, bruises, pain, warts, snakebite, fingernail inflammation, and onychomycosis [18, 19]. Additionally, previous research demonstrated that high levels of metals such as copper, zinc, chromium, and nickel could be accumulated by *Impatiens* species [20].

Due to the diversity of flowering and morphological characters in *Impatiens*, the phylogenetic relationships of *Impatiens* species remain uncertain [21]. *Impatiens* plants are characterized by zygomorphic flowers with substantial diversity and high levels of convergent evolution leading to variability in corolla color and morphology. The flowers are incredibly fragile, and most are coalesced and folded in dried specimens, making it difficult to separate and reconstruct different parts [22, 23]. Moreover, due to the semisucculent stems and many fleshy leaves, it is challenging to provide well-dried herbarium plant specimens [24]. Early research on *Impatiens* was primarily focused on a specific geographical area providing purely descriptive traditional taxonomy processing [25]. To date, the only global infrageneric molecular classification for *Impatiens* was performed based on plastid protein-coding genes *matK, rbcL,* and *trnK* and the intergenic regions *atpB-rbcL* and *trnL-trnF* [26, 27]. Additionally, nuclear ribosomal internal transcribed spacer (ITS) and inter-simple sequence repeat (ISSR) markers have been used to assess the genetic diversity of populations and to understand the phylogenetic and evolutionary relationships among *Impatiens* species [28]. However, all published data were based on relatively short sequences from material with obvious regional characteristics, and some species with diversified morphology were subject to taxonomic controversy due to unresolved phylogenetic relationships; thus, further studies and clarification are required [29]. For this reason, the present study is based on complete chloroplast genome sequences, which yield much better resolution for the reconstructing phylogenies [30].

Twelve complete chloroplast genomes of *Impatiens*, including six newly sequenced chloroplast genomes (*I. chlorosepala, I. fanjingshanica, I. guizhouensis, I. linearisepala, I. loulanensis* and *I. stenosepala*), from the karst area of China were assembled by using Illumina sequencing technology and combined with previously published complete Balsaminaceae chloroplast genomes [31]. The present investigation is a novel attempt to reveal the phylogenetic position and taxonomic status of *Impatiens* based on the whole chloroplast genome. The aims of this study were to (i) conduct comprehensive research on the *Impatiens* chloroplast genome, generating information on basic genome structure, codon usage, repetitive structure characteristics, and IR expansion; (ii) identify hotspot regions, microsatellite types, and comparative genomic divergence; and (iii) reconstruct and analyze the relationships of *Impatiens* species and determine the taxonomic status of *Impatiens* based on the complete chloroplast genomes.

## Results

### General features of *Impatiens*

**Fig. 1:**
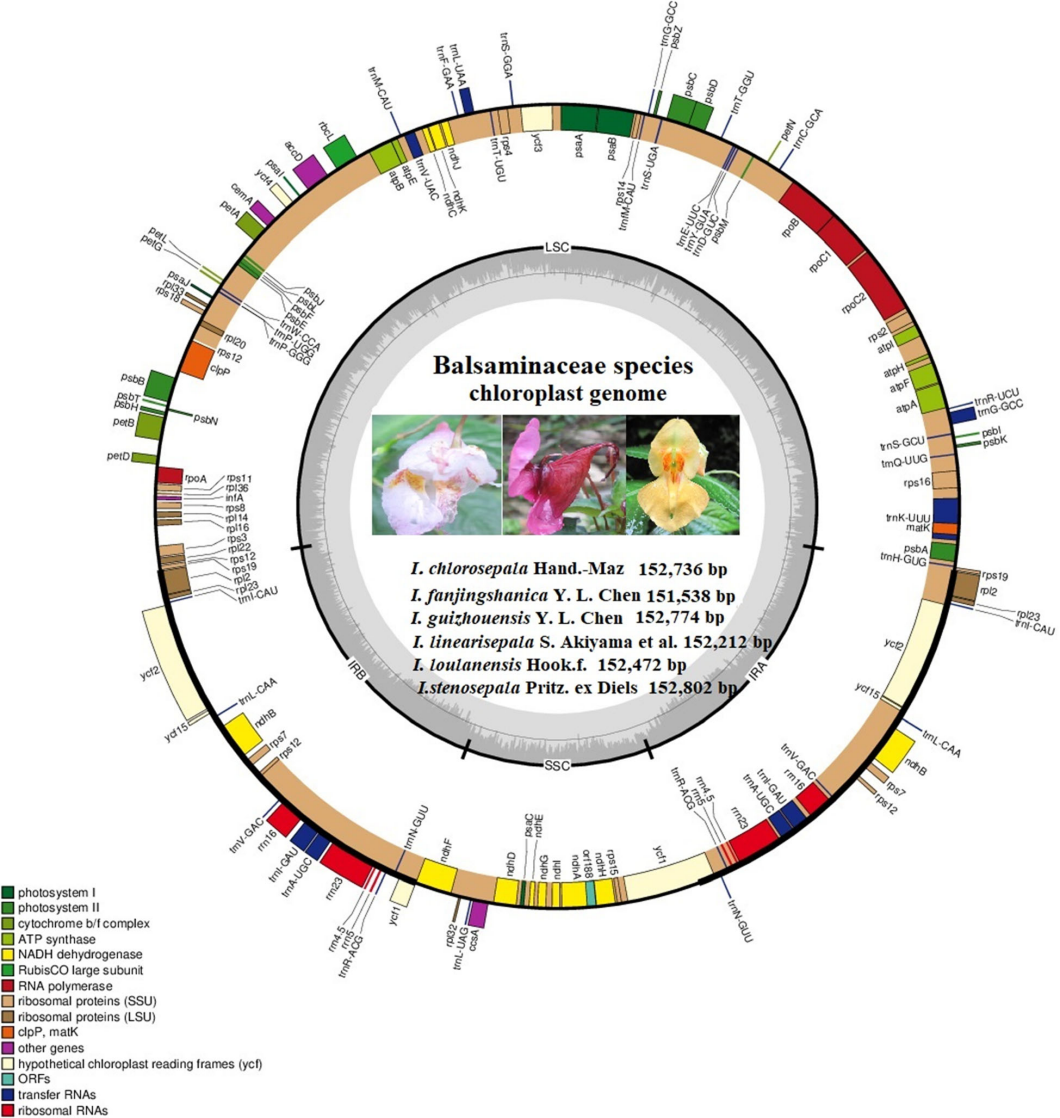
Chloroplast genome structure of *Impatiens* species (*I. chlorosepala, I. fanjingshanica, I. guizhouensis, I. linearisepala, I. loulanensis,* and *I. stenosepala*). Genes shown outside the circles are transcribed clockwise, while those drawn inside are transcribed counter clockwise. Genes are color-coded according to functional group (see the key at the down left). The positions of the long single-copy (LSC), short single-copy (SSC), and inverted repeat (IR: IRA and IRB) regions are shown in the inner circles

The genomic libraries generated 4.2–4.9 Gb of raw data, which were equivalent to 2.1–2.6 Gb of trimmed reads. After sequencing, cutting, and selecting reads, the 12 complete Balsaminaceae species chloroplast genomes ranged in size from 151,366 bp (*I. alpicola*) to 154,189 bp (*H. triflora*) (Table 1). The newly sequenced *Impatiens* chloroplast genome maps were provided in Fig. 1 and Supplementary Figs. S1-S6 (*I. chlorosepala, I. fanjingshanica, I. guizhouensis, I. linearisepala, I. loulanensis,* and *I. stenosepala*)*.* Similar to the pattern observed in other typical chloroplast genomes of angiosperms, the common feature of the complete chloroplast genomes consisted of four conjoined regions forming a circular molecular structure. The IRs were separated by LSC and SSC regions. In the chloroplast genomes of the family Balsaminaceae, the LSC region accounted for 54.47–55.04% of the total chloroplast genome, ranging from 82,247 bp (*I. alpicola*) to 84,865 bp (*H. triflora*); the SSC accounted for 11.37–11.73% of the total chloroplast genome, ranging from 17,309 bp (*I. linearisepala*) to 18,080 bp (*H. triflora*); and the IR accounted for 16.62–16.98% of the total chloroplast genome, ranging from 25,622 bp *(H. triflora*) to 25,773 bp (*I. chlorosepala*). In the newly sequenced chloroplast genomes of genus *Impatiens*, the LSC region accounted for 54.47–54.86% of the total chloroplast genome, ranging from 82,542 bp (*I. fanjingshanica*) to 83,508 bp (*I. linearisepala*); the SSC accounted for 53.58–58.27% of the total chloroplast genome, ranging from 17,309 bp (*I. linearisepala*) to 17,547 bp (*I. fanjingshanica*); and the IR accounted for 16.83–16.98% of the total chloroplast genome, ranging from 25,720 bp (*I. stenosepala*) to 25,773 bp (*I. chlorosepala*).

**Table 1:** Newly sequenced complete chloroplast genomes of *Impatiens* species

	***I. chlorosepala***	***I. fanjingshanica***	***I. guizhouensis***	***I. linearisepala***	***I. loulanensis***	***I. stenosepala***
Total length (bp)	152,763	151,538	152,774	152,212	152,472	152,802
GC (%)	36.7	36.9	37	37	36.7	36.9
LSC region length (bp)	83,740	82,542	83,572	83,508	83,460	83,626
GC (%)	34.3	34.6	34.8	34.8	34.4	34.5
SSC region length (bp)	17,477	17,547	17,662	17,309	17,541	17,739
GC (%)	29.5	29.4	29.9	30	29.6	29.8
IR region length (bp)	25,773	25,726	25,772	25,699	25,737	25,720
GC (%)	43.1	43.1	43	43	43	43.2
CDS length (bp)	79,562	79,689	79,941	79,533	79,650	79,581
GC (%)	37.2	37.2	37.4	37.3	37.1	37.2
rRNA length (bp)	9048	9048	9046	9048	9048	9048
GC (%)	55.1	55.1	55.1	55.2	55.1	55
tRNA length (bp)	2876	2872	2872	2872	2872	2884
GC (%)	52.4	52.6	52.7	52.5	52.6	52.6
Total genes	115	115	115	115	115	115
CDSs	81	81	81	81	81	81
tRNAs	30	30	30	30	30	30
rRNAs	4	4	4	4	4	4

Similar to most angiosperm chloroplast genomes, those of the Balsaminaceae species (except for *I. alpicola, I. pritzelii,* and *I. glandulifera*) encoded 115 distinct genes, including 81 protein-coding, 30 tRNA, and 4 rRNA genes (Supplementary Table S2). However, the *trnG-UCC* gene was annotated as a pseudogene in *H. triflora* compared to the other *Impatiens* species. The genes *psbN, trnK-UUU, trnL-UAA, trnP-GGG, ycf15* and *trnfM-CAU* were missing due to incorrect annotation in *I. glandulifera*. The pseudogene *orf188* was missing in *I. alpicola* and *I. pritzelii.* Thirteen genes (*ccsA, nahA, ndhD-I, orf188, psaC, rpl32, rps15,* and *trnL-UAG*) were not annotated in *I. alpicola.* The genes were classified into three groups based on their functions: (1) transcription and RNA genes, including four transcription genes (rpoA, rpoB, rpoC1*, and *rpoC2*), 20 ribosomal proteins, 4 ribosomal RNAs (*rrn4.5, rrn5, rrn16,* and *rrn23*), and 30 transfer RNAs; (2) photosynthesis-related genes (in the Rubisco, ATP synthase, Photosystem I, Cytochrome b/f complex, Photosystem II, Cytochrome c synthesis, and NADPH dehydrogenase groups); and (3) other genes, including four genes (*matK*, *cemA*, *accD*, and *clpP*) with known functions and three conserved reading frame genes (*ycf1, ycf2,* and *ycf15*) encoding proteins (Table 2 and Supplementary Table S1).

**Table 2:** List of genes in the chloroplast genomes of the *Impatiens* species

Function of Genes	Group of Genes	Gene Names
Photosynthesis-related genes	Rubisco	*rbcL*
	Photosystem I	*psaA, psaB, psaC, psaI, psaJ*
	Assembly and stability of Photosystem I	ycf3**, ycf4
	Photosystem II	*psbA, psbB, psbC, psbD, psbE, psbF, psbH, psbI, psbJ, psbK, psbL, psbM, psbN, psbT, psbZ,*
	ATP synthase	atpA, atpB, atpE, atpF*, atpH, atpI
	Cytochrome b/f complex	petA, petB*, petD, petG, petL, petN
	Cytochrome c synthesis	*ccsA*
	NADPH dehydrogenase	ndhA*, ndhB*(2), ndhC, ndhD, ndhE, ndhF, ndhG ndhH, ndhI, ndhJ, ndhK
Transcription- and translation-related genes	Transcription	rpoA, rpoB, rpoC1*, rpoC2
	Ribosomal proteins	rpl2*(2), rpl14, rpl16, rpl20, rpl22, rpl23(2), rpl33, rpl36, rps2, rps3, rps4, rps7(2), rps8, rps11, rps12*(2), rps14, rps15, rps16*, rps18, rps19(2)
RNA genes	Ribosomal RNA	*rrn4.5, rrn5, rrn16, rrn23*
	Transfer RNA	trnA-UGC(2), trnC-GCA, trnD-GUC, trnE-UUC, trnF-GAA, trnfM-CAU, trnG-GCC*, trnG-UCC, trnH-GUG, trnI-CAU*(2), trnI-GAU(2), trnK-UUU*, trnL-CAA(2), trnL-UAG, trnL-UAA*, trnM-CAU, trnN-GUU(2), trnP-UGG, trnQ-UUG, trnR-ACG(2), trnR-UCU, trnS-GCU, trnS-GGA, trnS-UGA, trnT-GGU, trnT-UGU, trnV-GAC(2), trnV-UAC*, trnW-CCA, trnY-GUA
Other genes	RNA processing	*matK*
	Carbon metabolism	*cemA*
	Fatty acid synthesis	*accD*
	Proteolysis	clpP**
Genes of unknown function	Conserved reading frames	*ycf1, ycf2(2), ycf15(2)*

(2) indicates that m (the number of repeat units) is 2; *Gene contains one intron; **Gene contains two introns

A total of 16 chloroplast genes had introns in the *Impatiens* species. Introns were missing in one of these genes in *I. piufanensis* (*rps16*) and *H. triflora* (*trnG-GCC* tRNA gene), respectively. The 16 genes could be classified into two groups according to their introns: group I included 14 genes with a single intron, and group II included two genes (*ycf3* and *clpP*) with two introns. Eleven of these intron-containing genes (*clpP, ycf3, trnv-UAC, rps12, trnK-UUU, rpoC1, petB, trnL-UAA, atpF, trnG-GCC,* and *rps16*) were in the LSC region, four genes (*tRNA-GAU, trnA-UGC, ndhB,* and *rpl2*) were in the IR region and only one gene (*ndhA*) was in the SSC region. The longest intron was within *trnK-UUU*, which ranged from 2488 bp (*I. loulanensis*) to 2548 bp (*I. guizhouensis*), and the exon of *rpoC1* was the longest. Moreover, *rps12* is a trans-splicing gene that was divided into 5′-*rps12* in the LSC region and 3′-*rps12* in the IR region (Table 2 and Supplementary Table S3).

### Differences in genome size

Among the 12 Balsaminaceae species, *I. alpicola* had the smallest chloroplast genome (151,366 bp), and *H. triflora* had the largest chloroplast genome (154,189 bp). Among the six newly sequenced species, *I. stenosepala* had the largest chloroplast genome (152,802 bp), whereas *I. fanjingshanica* had the smallest (151,538 bp). Except for *I. stenosepala* and *I. fanjingshanica*, the genome sizes of *Impatiens* species varied between 152,212 bp and 152,774 bp (Table 1). Except for *I. fanjingshanica*, the genome sizes of other Balsaminaceae species were larger than 152,000 bp (Supplementary Table S1). In the 12 Balsaminaceae species, the lengths of the protein-coding genes ranged from 79,533 bp (*I. linearisepala*) to 80,952 bp (*H. triflora*), and the length of the rRNAs totaled 9048 bp except in *I. guizhouensis*, *I. glandulifera*, and *H. triflora*, for which the lengths were 9046 bp, 9050 bp, and 9046 bp, respectively. The length of the tRNA genes added 2872 bp except in *I. chlorosepala, I. stenosepala, I. glandulifera,* and *H. triflora*, whose lengths added 2876 bp, 2884 bp, 2419 bp, and 2815 bp, respectively (Supplementary Table S1). The overall guanine-cytosine (GC) contents in the whole chloroplast genomes and the LSC, SSC, and IR regions were very similar among the species. The total GC content in the Balsaminaceae species ranged from 36.7 to 37%, with *I. chlorosepala* and *I. loulanensis* having the lowest GC content and *I. guizhouensis* and *I. linearisepala,* the highest (Table 1). The average GC contents of the LSC, SSC, and IR regions were 34.56, 29.7, and 43.0%, respectively (Table 1 and Supplementary Table S1).

### Codon usage

The most commonly used transcription initiation codon was ATG. The termination codons were UGA, UAG, and UAA. For the Balsaminaceae species (Supplementary Table S4), we found that the most abundant amino acid (AA) was leucine and that UUA had the highest relative synonymous codon usage (RSCU) value at approximately 1.92. Tryptophan was the lowest-frequency AA in the Balsaminaceae species. All AAs, except for methionine and tryptophan, had more than one synonymous codon. Among the AAs, leucine, arginine, and serine had six codons. The RSCU results indicated a bias toward A or T rather than G or C at the third codon position in the 12 Balsaminaceae species. *I. glandulifera* uses 30 different codons, which is lower than the expected usage at equilibrium (RSCU< 1). *H. triflora* used 36 codons more frequently than the rest of the *Impatiens* species, showing codon usage bias for 34 codons.

### Repeat structure analysis

**Fig. 2:**
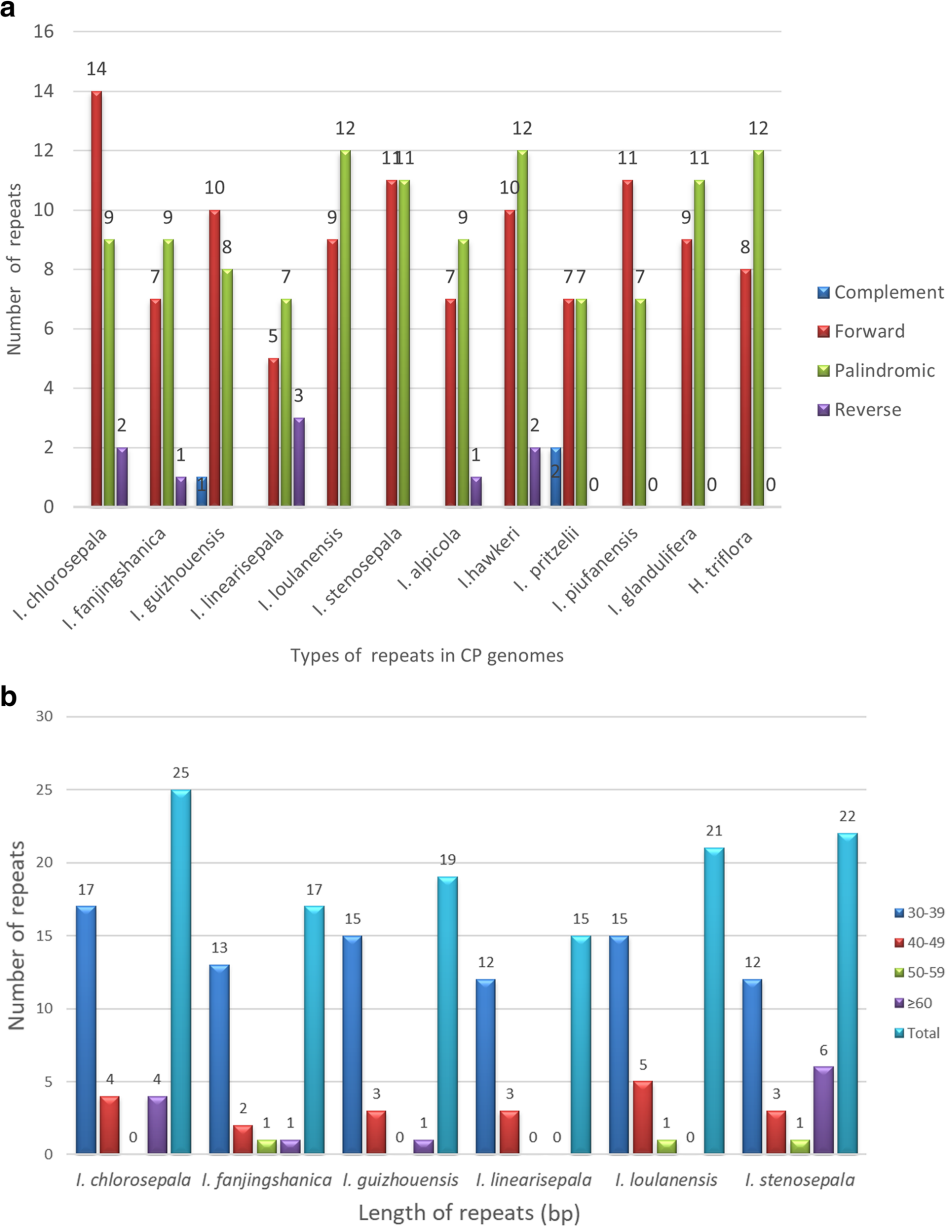
Repeated sequences in Balsaminaceae chloroplast genomes. (**A**) Total numbers of four repeat types in 12 Balsaminaceae chloroplast genomes. (**B**) Numbers of repeats sequences by length

Among the 12 Balsaminaceae species, 234 long repeats of four types (forward, complement, reverse, and palindromic) were identified using REPuter (Supplementary Table S5). The most common repeat types were forward and palindromic repeats. Complement repeats were identified only in *I. guizhouensis* and *I. pritzelii*; reverse repeats were found in *I. chlorosepala, I. fanjingshanica, I. linearisepala, I. pritzelii,* and *I. hawkeri*. Most copy lengths were in the range of 30–40 bp (Fig. 2B). The species with the most significant number of repeats were *I. chlorosepala,* with 25 repeats, comprising 14 forward, 9 palindromic, and 2 reverse repeats. *I. linearisepala,* which had the smallest number of repeats, had 5 forward, 7 palindromic, and 3 reverse repeats (Fig. 2A). The greatest numbers of forward, complement, and reverse repeats were found in *I. chlorosepala* (14), *I. pritzelii* (2), and *I. linearisepala* (3), respectively.

### Simple sequence repeat analysis

**Fig. 3:**
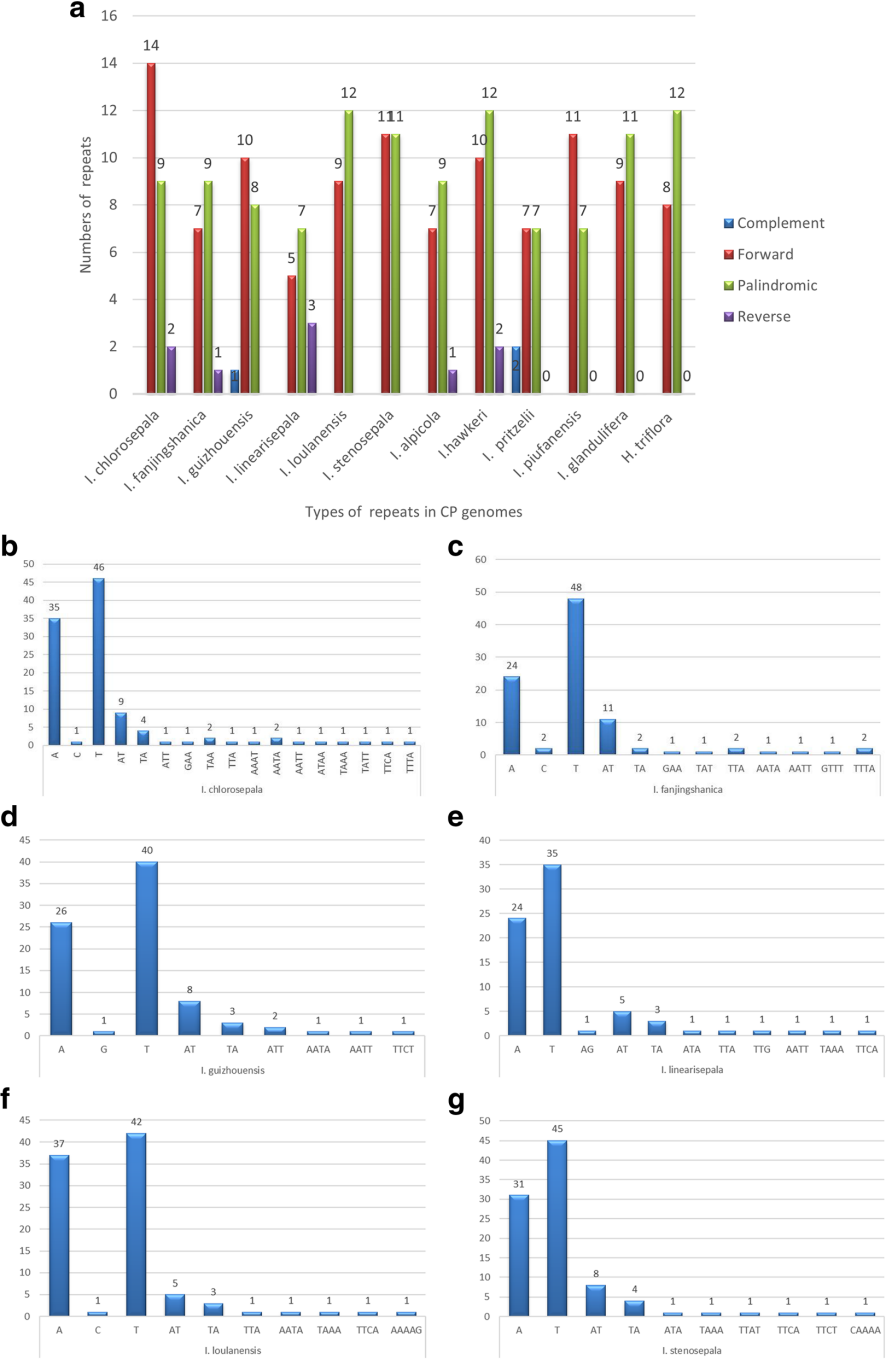
SSR locus analysis of 12 Balsaminaceae chloroplast genomes. (**A**) Numbers of different SSR types detected in the 12 genomes. (**B-G**): Frequencies of identified SSR motifs in different repeat class types

Simple sequence repeats (SSRs), also called microsatellites, are widely used as molecular markers and play a significant role in plant identification and classification. The 51–109 SSRs examined for the Balsaminaceae species ranged in size from 10 to 20 bp. Six types of SSRs were found (Fig. 3A and Supplementary Table S6). Only *H. triflora* had hexanucleotide repeats, whereas *I. loulanensis, I. stenosepala,* and *H. triflora* had pentanucleotide repeats. The number of mononucleotide repeats ranged from 33 (*H. triflora*) to 82 (*I. chlorosepala*), followed by dinucleotides, ranging from 5 (*I. hawkeri*) to 13 (*I. chlorosepala, I. fanjingshanica,* and *I. glandulifera*) (Fig. 3B-G). Therefore, mononucleotide and dinucleotide repeats may play a more significant role than other types of repeats in genetic variation.

Mononucleotide repeats were more abundant in the six newly sequenced chloroplast genomes, with A/T repeats being the most highly represented repeats, whereas poly C/G repeats were relatively rare. Poly C/G repeats were found only in *I. chlorosepala, I. fanjingshanica, I. guizhouensis,* and *I. loulanensis.* Moreover, the number of mononucleotide repeats ranged from 24 (*I. fanjingshanica* and *I. linearisepala*) to 37 (*I. loulanensis*), with the number of T mononucleotide repeats ranging from 35 (*I. linearisepala*) to 48 (*I. fanjingshanica*) (Fig. 3B-G).

Among the dinucleotide repeats, the AT/TA motif was the most abundant. In the newly sequenced chloroplast genomes, SSR analysis showed that *I. chlorosepala* had the highest number of SSRs (109), while *I. linearisepala* had the lowest (74). Trinucleotide (ATT, GAA, TAA, TTA, TAT, ATA, and TTG) and tetranucleotide (AAAT, AATA, AATT, ATAA, TAAA, TATT, TTCA, TTTA, GTTT, and TTCT) motifs were identified. Among the newly sequenced chloroplast genomes, pentanucleotide (AAAAG and CAAAA) repeats were found only in those of *I. loulanensis* and *I. stenosepala*.

### Comparison of genome structures

**Fig. 4:**
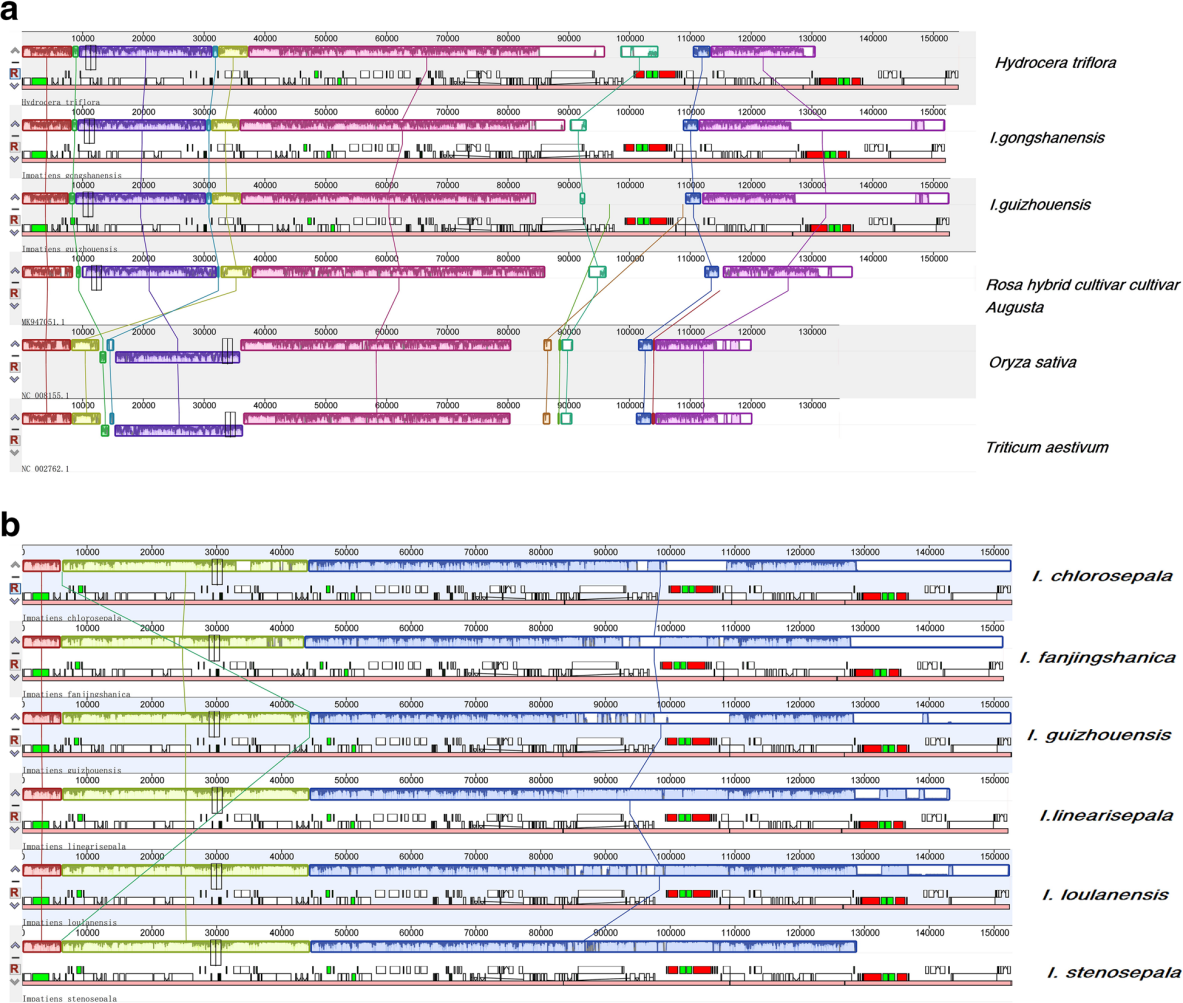
Mauve alignment. (**A**) Two rearrangements concerning the dicot plastome with LSC and IRB intermolecular recombination. (**B**) Mauve alignment of six Balsaminaceae plastomes revealing no interspecific rearrangements

The structure and size of the chloroplast genome can change based on the evolutionary and genetic backgrounds. Collinearity detection was used to analyze and compare the chloroplast genomes. Mauve alignment of plastomes showed that the plastome structure of *Impatiens* was similar to that of the dicot *Rosa* (MK947051) (Fig. 4A)*.* However, on the basis of a comparison with the monocots *Triticum aestivum* (NC002762) and *Oryza sativa* (NC008155), the monocot and dicot structures were derived from intermolecular recombination events (Fig. 4A). There were no interspecific or intraspecific rearrangements within the six species, which revealed that all genes (including rRNA, tRNA, and protein-coding genes) in the Balsaminaceae were conserved and arranged in the same order (Fig. 4B); this also applied to the optimal collinearity between *Impatiens* subgenera, as there were no gene rearrangements. Moreover, compared with the genome structure and gene sequence of *H. triflora*, those of the *Impatiens* subgenera were similar.

### Comparative analysis of genomic divergence and genome rearrangement

**Fig. 5:**
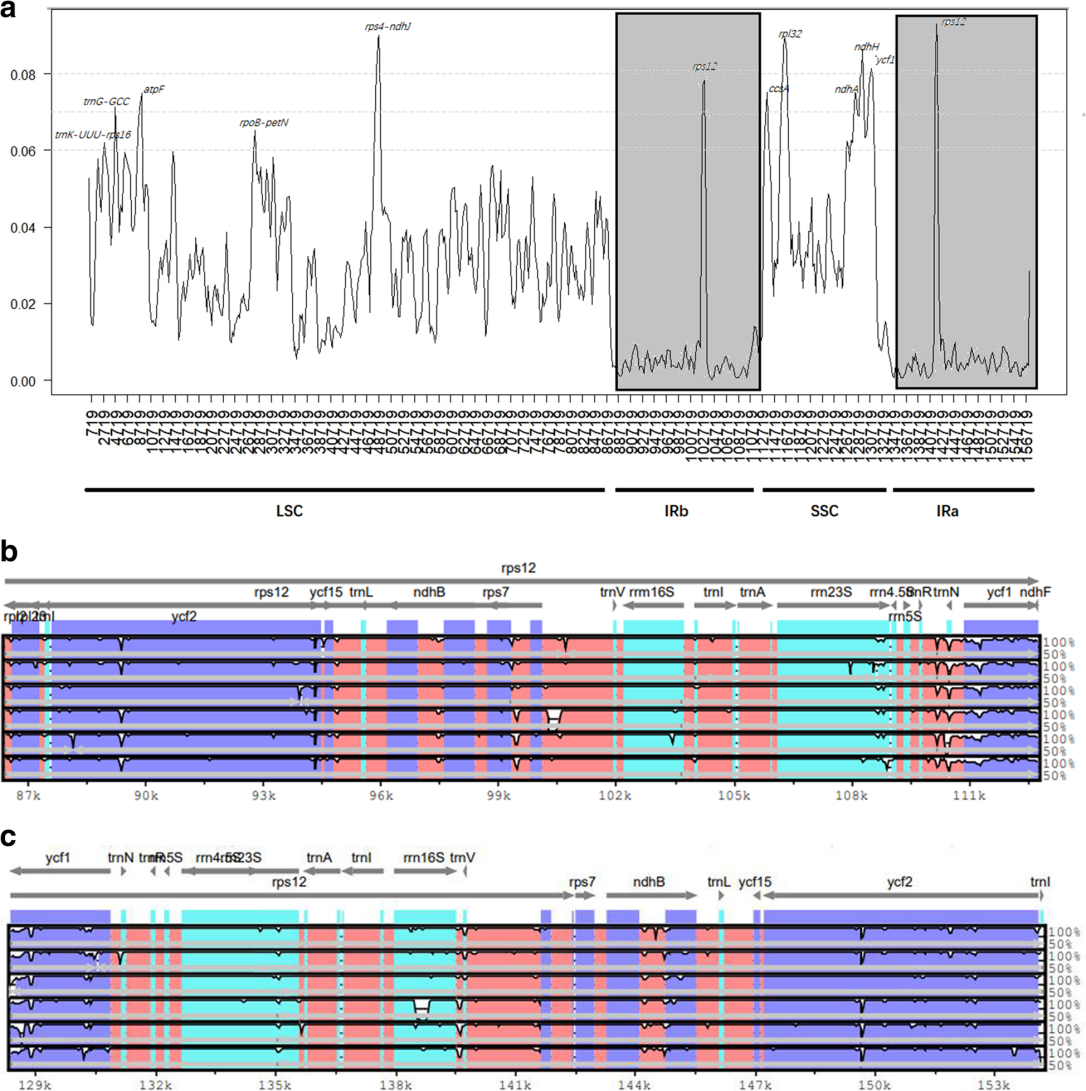
(**A**) Sliding window analysis of the newly sequenced chloroplast genomes of Balsaminaceae species. (**B**) The sequence divergence from 87,000 bp to 111,000 bp visualized by the mVISTA program. The vertical scale indicates percent identity, ranging from 50 to 100%. (**C**) The sequence divergence from 129,000 bp to 153,000 bp visualized by the mVISTA program

A comparative analysis of the whole chloroplast genome between *H. triflora* and the other *Impatiens* species was conducted by using mVISTA software and DnaSP to detect hypervariable regions and construct sequence identity plots (Fig. 5A). The comparison showed that the numbers and sequences of genes in the IR regions were relatively conserved and less divergent than those in the LSC and SSC regions (Fig. 5B and C). Among the protein-coding genes, *matK, psbK, petN, psbM, atpE, rbcL, accD, psaL, rpl16, rpoB, ndhB, ndhF, ycf1,* and *ndhH* contained highly divergent regions (Fig. 5A). For the intergenic regions, *atpH-atpI, trnC-trnT, rps3-rps19*, and *ndhG-ndhA* were the most variable. In the LSC region, the *psbK-psbI, atpI*, and *rps4-trnF* genes showed some sequence divergence in *I. piufanensis, I. glandlifera,* and *H. triflora*. The three genes *ndhF, ycf1*, and *ndhH* were detected in the SSC region. *rpl32-trnN* showed the highest variation among the hypervariable regions, and the *ycf1* gene was the most divergent. Compared with those of *H. triflora*, the large copies of the *trnl-trnN* and *trnA-trnL* loci in the chloroplast genomes of *I. fanjingshanica, I. guizhouensis,* and *I. loulanensis* were absent.

### Sequence divergence and mutational hotspots

**Fig. 6:**
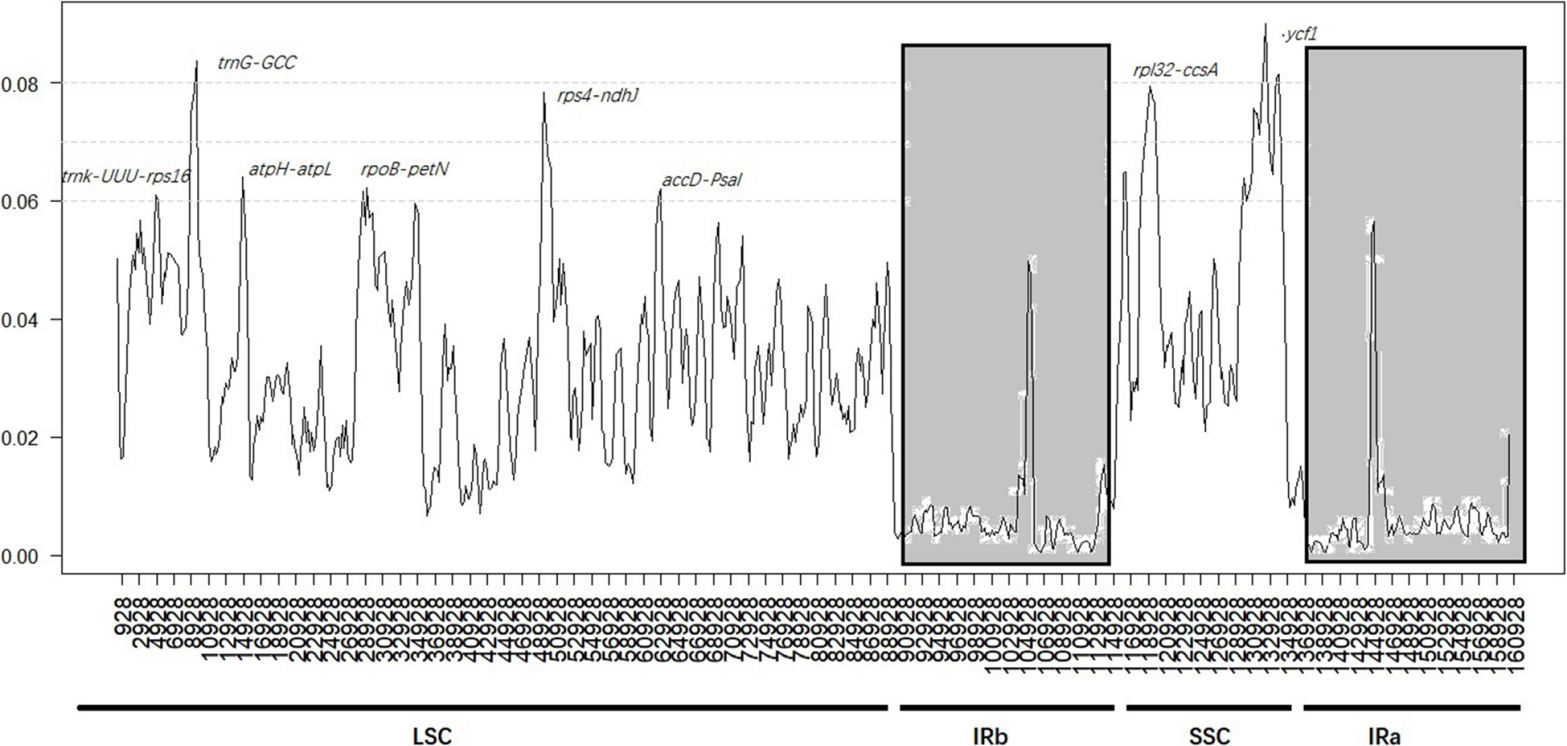
Sliding window analysis of the chloroplast genomes of 12 Balsaminaceae species. Window length: 2000 bp; step size: 200 bp. X-axis: the position of the midpoint of a window. Y-axis: the nucleotide diversity of each window

We compared nucleotide diversity (π) values in DnaSP 5.1 to determine the divergence hotspot regions in 12 Balsaminaceae species. This analysis indicated that the variation in the LSC and SSC regions was much higher than that in the IR regions (Fig. 6). The highest π values were observed for *ycf1* (0.17) and *trnG-GCC* (0.13). Six mutational hotspots that exhibited markedly higher π values (> 0.06) in the LSC and SSC regions were *trnk-UUU-rps16, trnG-GCC, atpH-atpL, rpoB-petN, rps4-ndhJ,* and *accD-psal,* whereas in the SSC region, there were three hotspots (*ndhF, rpl32-ccsA,* and *ycf1*) with values above 0.06. Similarly, we determined the average pairwise sequence divergence among newly sequenced *Impatiens* species. The π values of these 140 regions ranged from 0.0% (*rrn16*) to 9.3% (*rps12*). The *rps12* gene showed the highest average sequence divergence (0.93), followed by *rpl32* (0.91) and *rps4-ndhJ* (0.90) (Fig. 6 and Supplementary Table S7). In contrast, the π values of the six newly sequenced species were higher than those of the other 12 Balsaminaceae species. Therefore, these coding regions and noncoding genes may provide stronger signal for resolving the low-level phylogeny and phylogeography of species in Balsaminaceae.

### Contraction and expansion of inverted repeats

**Fig. 7:**
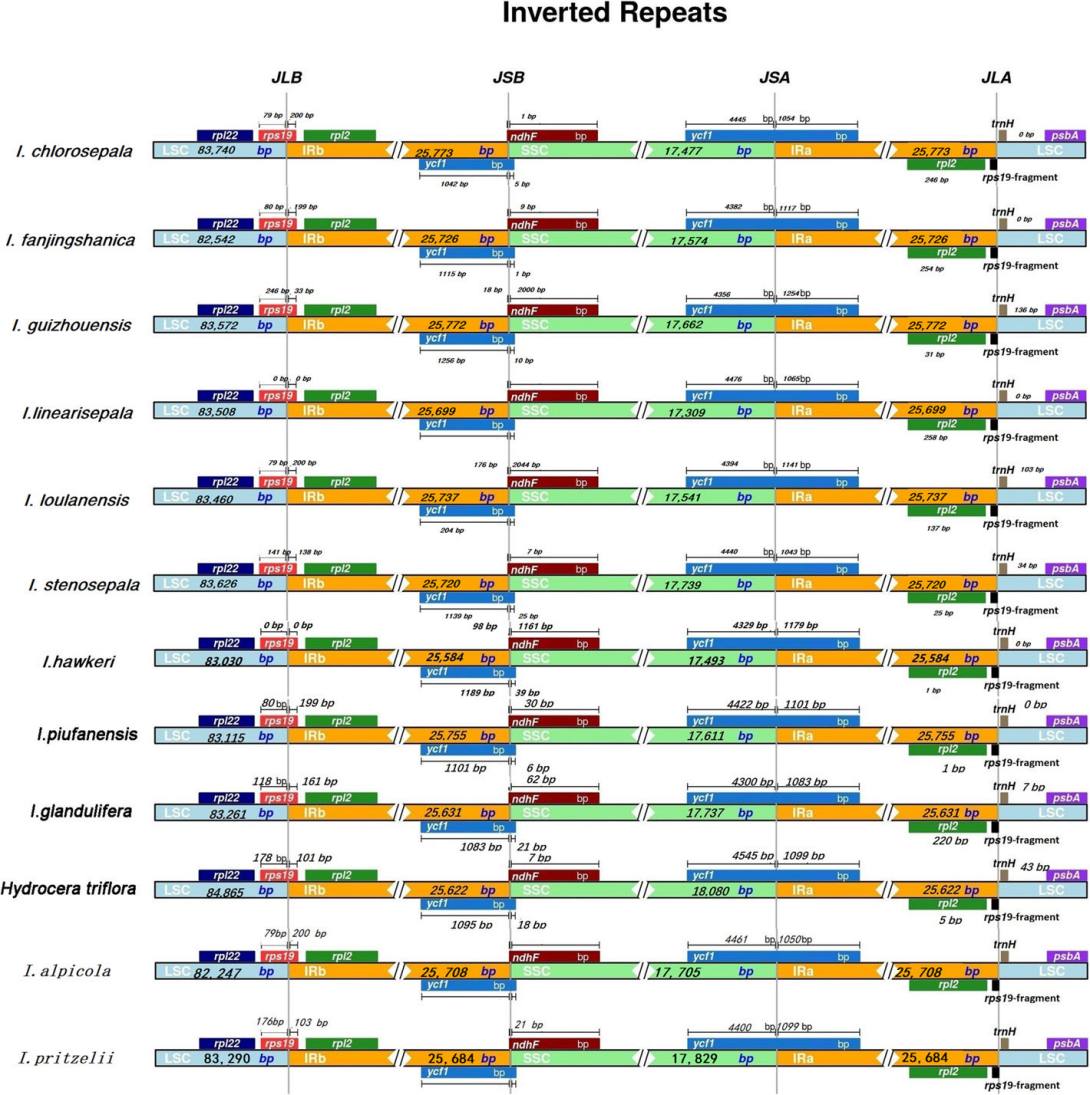
Comparison of the borders of the large single-copy (LSC), small single-copy (SSC) and inverted repeat (IR) regions among 11 chloroplast genomes. The numbers above the gene features indicate the distance between the ends of genes and the border sites

Genome structure and the number and sequence of genes were highly conserved among the 12 Balsaminaceae species. However, the contraction and expansion of IR boundaries changed in terms of structure and size. In the 12 Balsaminaceae species, we localized *ycf1* to the IRA-LSC boundary, the IRs of *I. chlorosepala* were the longest (25,773 bp), and those of *H. triflora* were the shortest (25,622 bp). The LSC-IRB junctions were embedded in the *rps19* genes. The length of *rps19* in the LSC region varied from 0 to 246 bp. However, the overlap between *rps19* and the IRB region varied from 0 to 200 bp. The IRB-SSC junction was located adjacent to genes *ycf1* and *ndhF*. In all species except for *I. linearisepala*, this junction adjoined the end of *ycf1* from 0 to 1256 bp, and the distance between *ycf1* and the IRB-SSC junction in *I. linearisepala* was 204 bp. Overlap between the *ndhF* and *ycf1* genes was detected in *I. guizhouensis, I. linearisepala,* and *I. hawkeri*, where *ndhF* expanded into the IRB region by 18 bp, 176 bp, and 98 bp, respectively (Fig. 7).

In the other species, the distances between *ndhF* and the IRB-SSC junction varied from 1 to 2000 bp. The SSC-IRA junction was located in the pseudogene *ycf1,* which covered the IRA and SSC regions. The length of the pseudogene *ycf1* in the SSC region varied from 4356 to 4891 bp. However, the overlap between the pseudogene *ycf1* and the IRA region varied from 810 to 1254 bp. The IRB/SSC and SSC/IRA regions were variable. The *rps19-psbA* coding region extended into the boundaries of the LSC/IRA regions in all species except *I. piufanensis, I. glandulifera,* and *H. triflora*, in which the *rps19* gene was missing from the junction of the LSC/IRB regions. However, the length of *rps19* in the LSC region varied from 0 to 136 bp. In contrast, the lengths of *rps19* in the IRB regions of *I. guizhouensis* and *I. stenosepala* were 31 and 137 bp, respectively. Among the six newly sequenced species, *I. chlorosepala* and *I. linearisepala* harbored the longest (25,773 bp) and shortest (25,699 bp) IR regions, respectively.

### Phylogenetic analyses within Balsaminaceae species

We used Maximum likelihood (ML) and Bayesian inference (BI)-based phylogenetic trees to explore the taxonomic positions and evolutionary relationships of Balsaminaceae species based on the complete chloroplast genomes (Supplementary Table S8). According to the APG IV classification for the orders and families of flowering plants, we selected six families belonging to Ericales, namely, Ebenaceae, Styracaceae, Actinidiaceae, Theaceae, Primulaceae, and Balsaminaceae. Species from families Saxifragaceae and Rosaceae were selected as outgroups due to their closer distances to Ericales as well as ornamental and horticultural value. The 12 Balsaminaceae species included those with published plastid genomes (*I. piufanensis, I. glandlifera, I. hawkeri, I. alpicola, I. pritzelii,* and *H. triflora*) and six newly sequenced species (*I. chlorosepala, I. fanjingshanica, I. guizhouensis, I. linearisepala, I. loulanensis,* and *I. stenosepala*).

**Fig. 8:**
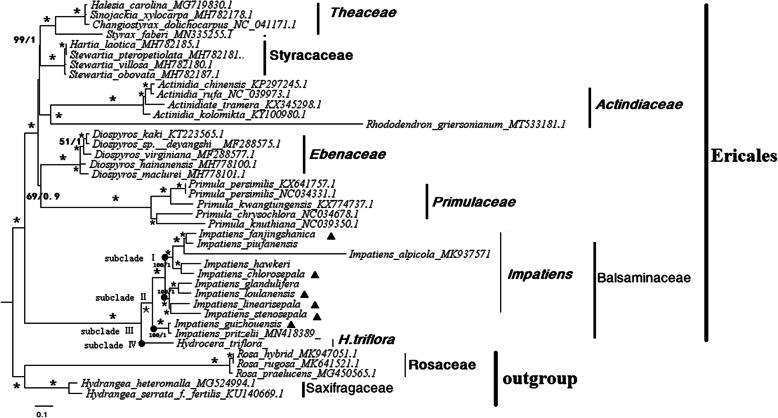
Phylogenetic tree based on whole chloroplast genome sequences from 12 Balsaminaceae species and 28 other related species displaying maximum likelihood bootstrap support (MLBS) values and Bayesian posterior probabilities (PPs). The ML topology is shown with MLBS values/Bayesian PPs given at each node. Asterisks indicate that PP = 1 and MLBS = 100%. Black dots indicate the four main lineages of *Impatiens* species. Black triangles indicate the chloroplast genomes of the six *Impatiens* species newly determined in this study

The ML and BI reconstructed topologies were highly supported, and the five selected families (Primulaceae, Actinidiaceae, Theaceae, Ebenaceae, and Styracaceae) other than Balsaminaceae formed five monophyletic groups. The genera *Hartia* and *Stewartia* of family Ebenaceae were clustered into a clade, whereas Theoideae consisted of *Actinidia* and *Rhododendron*. Only three nodes (Primulaceae, Theaceae, and Ebenaceae) had bootstrap values under 90% in the ML tree (Fig. 8). The remaining nodes had support values of 100%. Only two nodes (Primulaceae and Actinidiaceae) had posterior probability values under 0.9 in the BI tree. The remaining nodes had support values of 1 (Fig. 8).

Phylogenetic reconstruction using ML and BI (Fig. 8 and Additional File 2: Fig. S5) divided all Balsaminaceae species into four main lineages (I to IV) with maximal support (PP = 1, BS = 100%). Lineage I contained five *Impatiens* species (*I. chlorosepala, I. hawkeri, I. alpicola, I. piufanensis*, and *I. fanjingshanica*). Within this lineage *I. fanjingshanica* and *I. piufanensis* were sister species. Lineage II contained *I. stenosepala, I. linearisepala, I. loulanensis* and *I. glandulifera*. Within this lineage, *I. glandlifera* and *I. loulanensis* were sister to *I. linearisepala* and *I. stenosepala* with the species displaying the most similar morphological characteristics clustering together. Lineage III had only two species (*I. guizhouensis* and *I. pritzelii*). Lineage IV contained *H. triflora.*

## Discussion

### Chloroplast genome structure

Twelve complete chloroplast genomes of Balsaminaceae were compared and found to include 102–115 genes, including 69–81 protein-coding genes, 25–30 tRNAs, and 4 rRNAs. The chloroplast genomes of Balsaminaceae species exhibit a typical quadripartite structure consisting of two IR regions (LSC and SSC fragments) [32, 33]. The chloroplast genes of Balsaminaceae were similar in size (there was a maximum 1436 bp difference in length between the *Impatiens* species) and composition (overall GC contents varied between 34.3 and 34.8%). Among the plastomes, that of *H. triflora* was the largest (154,189 bp). Compared with that of *H. triflora*, the plastomes of the *Impatiens* species were reduced in size by approximately 1387–2823 bp. In the most reduced plastome (151,538 bp) of *I. fanjingshanica*, contraction and expansion of IR boundaries were observed, suggesting that these processes are partly responsible for plastome downsizing in Balsaminaceae species. Potential *trnG-UCC* genes were annotated in all genomes of the *Impatiens* species but not in that of *H. triflora*. Converserly, the *pbf1* gene was annotated only in *I. glandulifera*. Approximately 13 photosynthesis-related genes (*ccsA, nadA, ndhD-I, orf188, psaC, rpl32, rps15,* and *trnL-UAG*) were missing due to incorrect annotation in *I. alpicola*. The GC content of *I. chlorosepala* was found to be lower than that of the other species (Table 1). The GC content in the IR regions was much higher than that in the LSC and SSC regions in all Balsaminaceae species. The rRNA and tRNA genes had high GC contents (52–55%). Usually, a higher GC content indicated a more stable genome sequence. These data strongly showed that chloroplast genomes differ within the same family [33]. However, the basic structures and contents of the genomes were generally similar.

### IR expansion and contraction

In most cases, gene gain or loss in chloroplast genomes is due to the contraction or expansion of genome regions. The presence of the pseudogene *ycf1* could be the result of such events. This was apparent in the plastomes of *I. chlorosepala, I. guizhouensis*, and *I. loulanensis,* where the IRs were much longer. Interestingly, the chloroplast LSC borders in *I. linearisepala* and *I. guizhouensis* were quite different from those in the other Balsaminaceae species, as the *ndhF* gene extended into the IRs and SSC region. In *I. guizhouensis* and *I. stenosepala*, the *rps19* gene extended into the IRs and LSC region. The LSC region in *I. fanjingshanica* was shorter than that in the other 11 chloroplast genomes.

### Repetitive sequence and simple sequence repeat analyses

Analysis of various chloroplast genomes showed that repetitive sequences were essential for inducing indels and substitutions [34]. These sequences were not only play an important role in the rearrangement and stabilization of the chloroplast genome sequence but also affect the copy number differences among species [35]. We identified a total of 234 repeats in Balsaminaceae, falling into four different repetitive categories (Supplementary Table S5). Among all species, the most common types corresponded to palindromic repeats, which occurred 114 times (49.59%), followed by forward repeats (108 instances, 46.34%). Complement repeats were identified only in *I. guizhouensis* and *I. pritzelii*. Reverse repeats were found only in *I. chlorosepala, I. fanjingshanica, I. linearisepala, I. alpicola,* and *I. hawkeri.*

SSRs have been recognized as a primary source of molecular markers because they have a high polymorphism rate and abundant variation at the species level. Moreover, SSRs are useful for detecting genetic diversity and polymorphisms at the population, intraspecific, and cultivar levels, as well as for distinguishing species [36, 37]. A total of 51–109 SSRs were identified, with an overall length ranging from 3 to 10 bp. Additionally, mononucleotide SSRs were detected in all Balsaminaceae species with the highest frequency, providing ample markers for phylogenetic analysis. The number of SSRs in *H. triflora* was lower than that in *I. chlorosepala*. Poly (A)/(T) SSRs are usually more common than other SSR repeat types, whereas poly (C/G) repeats are relatively rare. We identified only hexanucleotide SSRs (ATTGGG) in *H. triflora* and poly G SSRs in *I. guizhouensis*. We also identified SSR repeat units (TAAA/TTTA) unique to *I. chlorosepala* and *H. triflora.* Most chloroplast SSRs were observed in noncoding regions and were short mononucleotide tandem repeats, and they commonly showed intraspecific variation in repeat numbers [38]. Due to slippage of DNA strands, repeated loci, pairwise sequence divergence, and highly divergent regions were detected, indicating that the present findings will be useful for investigating genetic diversity levels and genomes presenting a high mutation rate.

### The utility of Plastomes in Phylogenomics and DNA barcoding

Divergence hotspots are usually used as evidence for species authentication and to provide phylogenetic information. Moreover, the IR regions show lower sequence divergence than the SSC and LSC regions. The noncoding regions and coding regions are less similar in angiosperm chloroplast genomes than in other genomes [39]. The following genes, *trnk-UUU-rps16, trnG-GCC, atpH-atpL, rpoB-petN, rps4-ndhJ, accD-psal, ndhF, rpl32-ccsA,* and *ycf1* were detected as the most divergent. Moreover, two regions (*trnG-GCC* and *ycf1*) showed high levels of variation both within all the Balsaminaceae species and within the newly sequenced species (π > 0.8%). In the newly sequenced species, noncoding regions, such as the *psbK-psbI, trnT-GGU-psbD, ycf4-cemA, rpl36-rps8, rpoB-trnC-GCA, trnP-UGG-psaJ, trnT-UGU-trnL-UAA, trnK-UUU-rps16,* and *trnQ-UUG* genes, possessed high variability and thus represented potential molecular marker.

### Phylogenomic validation

Balsaminaceae is considered to be a taxonomically controversial and complex family at both the morphological and molecular levels owing to their similar morphology and wide distribution areas of its constituent species [40]. Various analyses of the whole chloroplast genome have revealed that it contains sufficient informative loci for resolving molecular evolution and phylogenetic relationships within families and genera. The first molecular phylogeny of *Impatiens* was published by Fujihashi et al. [41]. However, the limited taxon samples and using of a distant outgroup (*Tropaeolum* in Tropaeolaceae) resulted in limited resolution of the phylogenetic relationships within the family. Based on nuclear ribosomal ITS and *atpB-rbcL*, phylogenetic studies of 111 Balsaminaceae species provided, new insights, such as *Impatiens* colonizing areas from Southwest China to the African continent in three separate diversification events [42]. Subsequently, based on plastid, combined plastid and nuclear or combined plastid and pollen data, *Impatiens* species were further analyzed [43]. Although these results have laid an important foundation for the identification and classification of Balsaminaceae species, all previous published data were based on relatively short sequences from material with obvious regional characteristics, and the numbers of nuclear/chloroplast genes were somewhat low [44], which limited phylogenetic conclusions. Therefore, the results were too conflicting to provide sufficient information for elucidating the phylogenetic and evolutionary relationships among Balsaminaceae species.

Phylogenetic reconstruction using ML and BI recovered Balsaminaceae sister to all other studied families of Ericales. Nodes received high support values (PP = 1, BS = 100%) (Fig. 8), and were highly congruent with those recovered in previous studies. Witihin Balsaminaceae, four major lineages were recovered in agreement with Yu [28], who proposed a new classification of *Impatiens* based on morphological characteristics and combined sequence data from three genetic regions, including nuclear ribosomal ITS and plastid *atpB-rbcL* and *trnL-F* molecular datasets. This classification divided *Impatiens* into two subgenera (*Clavicarpa* and *Impatiens*) and seven sections of the subgenus *Impatiens* [28]. *I. stenosepala* belongs to the Semeiocardium section, which is characterized by the lack of peduncle or only a very short peduncle, fusion of the lower lobes of the united lateral petals on each side, obconic capsules, and brick-shaped seeds [45]. Here, we found that *I. stenosepala* was sister to *I. linearisepala* and both are four-carpellate. *I. chlorosepala* belongs to the Uniflorae section, which is characterized by short fusiform capsules and a lack of peduncles. *I. loulanensis* has lower funnelform sepals and petioles without basal glands [46]. Compared to those of other related *Impatiens* species, the genome structure of *I. guizhouensis* was more similar to that of *H. triflora*. According to the reconstructed phylogeny, the chloroplast genome structure of *I. guizhouensis* represented the ancestral state of the Balsaminaceae family.

The morphological features of *H. triflora* were as follows: leaves alternate, linear-lanceolate, sessile, sepals 4, unequal length, stamens 5, ovaries 5, and ovules per locule 2–3 [47]. The characteristics of *Impatiens* were as follows: valgus lip with single leaves, spirally arranged, opposite or whorled, stalked or sessile, sepals 3, sparsely 5, lateral sepals free or connate, entire or toothed, pistil composed of 4 or 5 carpels, ovary upper, compartments 4 or 5, each with 2 to many anatropous ovules [46]. The morphology of *I. guizhouensis* was similar to that of *H. triflora*, despite *I. guizhouensis* belongs to the genus *Impatiens,* suggesting the retention of ancestral character states. Overall, using the complete chloroplast genome may be suitable for understanding the mechanisms of evolution and substantially increasing the power to discriminate species in evolutionary lineages [48–50].

## Conclusions

Comparative analyses involving 12 Balsaminaceae plastomes provided important new insights into plastome structure and evolution. Within the karst regions inhabited by *Impatiens* species, plastomes showed highly similar basic structures, sizes, GC contents, and gene numbers, orders and functions. However, contraction or expansion of IRs, sequence divergence and mutational hotspots, and a number of duplicated genes in the IRs were detected. Our results revealed highly variable regions that can be used as potential markers for species identification and phylogenetic inference. Additionally, ML- and BI-based phylogenomic analysis of chloroplast genome sequences yielded more accurate phylogenetic relationships within the Balsaminaceae and might provide valuable genomic resources for systematic evolutionary analyses of the family. Therefore, whole-chloroplast genomics is useful for species identification, taxonomic clarification, and genomic evolutionary analysis. Further research on the relationships within Balsaminaceae should incorporate morphology and genome-wide analyses to enhance our understanding of evolution.

## Methods

### Ethical statement

No specific permits were required for the collection of specimens for this study. This research was carried out in compliance with the relevant laws of China.

### Materials and DNA extraction

In total, 12 individuals of Balsaminaceae species were included (Supplementary Table S1). Prof. Haiquan Huang collected and identified all newly sequenced plants in the karst area of Guizhou, Yunnan and Guangxi, and data for an additional six species were downloaded from GenBank. All voucher specimens were deposited in the Plant Laboratory of Southwest Forestry University, Kunming, Yunnan, China (Table 3). Fresh leaves were collected and immediately stored in liquid nitrogen. We extracted genomic DNA by using the Tiangen DNA Reagent Extraction Kit (Tiangen Biotech, Beijing, China) [49]. Approximately 5–10 μg of genomic DNA was checked using spectrophotometry, and DNA integrity was examined by electrophoresis on a 1.5% agarose gel [51].

**Table 3:** List of basic information for the *Impatiens* specimens

Specimen	Altitude	Latitude and Longitude	Location	Voucher Specimen
*I. chlorosepala*	821 m	N22°58′964″ E106°75′696″	Orchid Valley Park, Pingxiang City, Congzuo City, Guangxi Province, China	SWFU-IBLE20161008
*I. fanjingshanica*	540 m	N27°53′8″ E108°47′8″	Ganzitang, Tongren City, Guizhou Province, China	SWFU-IBFJS20171030
*I. guizhouensis*	870 m	N27°52′6″ E108°45′6″	Fanjingshan Xiaochuanwan, Tongren City, Guizhou Province, China	SWFU-IBGZ20171030
*I. linearisepala*	1320 m	N23°13′285″ E104°85′667″	Malipo Laoshan Nature Reserve, Wenshan City, Yunnan Province, China	SWFU-IBXE20180928
*I. loulanensis*	1741 m	N26°10′249″ E104°35′593″	Luoduo Village, Yuni Township, Pan County, Liupanshui, Guizhou Province, China	SWFU-IBLN20161013
*I. stenosepala*	730 m	N27°51′55″ E108°45′33″	Fanjing Mountain Erdaoguai, Tongren City, Guizhou Province, China	SWFU-IBZE20171030

### Illumina sequencing, assembly, and annotation

Using an Illumina MiSeq sequencer (PE150 reads), libraries were constructed based on purified genomic DNA and sequenced. The quality of paired-end Illumina reads was assessed with FastQC, and Bowtie v2.2.6 software was used with the default settings to select trimmed reads that corresponded to the plastid, using the plastome of *I. piufanensis* as a reference [50]. The Contigs were assembled using SPAdes 3.6.1. The assemblies were manually corrected using GetOrganelle version 1.6.2 with default settings [52]. Each assembled chloroplast genome was annotated with GeSeq and Dual Organellar Genome Annotator (DOGAM), and the start and stop codon positions were further searched by homologous gene identification [53, 54]. In addition, the position of intron-exon junctions in the protein-coding genes, rRNAs, and tRNAs was confirmed with the BLASTN and tRNAscan v1.23 programs [55]. The genes were manually corrected when necessary and verified using Geneious R8.0.2 by realignment with references [56]. Physical circular chloroplast genome maps were generated by OGDrawV1.2 software [57]. Protein-coding genes were detected by comparison with the reference species *I. glandulifera* (GenBank MK358447) and *I. piufanensis* (GenBank MG162586). The GC content was calculated with Geneious R8.0.2.

### Repeat sequence and simple sequence repeat analyses

The size and location of repeat sequences (forward, palindromic, reverse, and complement repeats) were identified by REPuter [58] with the following settings: sequence identity was 90%, the Hamming distance was 3, and the minimum repeat size was 30 bp. Online MISA software (http://pgrc.ipk-gatersleben.de/misa/misa.html) was used to detect SSRs with minimum repeat number settings of 10, 5, 4, 4, 4, and 4 for mononucleotides, dinucleotides, trinucleotides, tetranucleotides, pentanucleotides, and hexanucleotides, respectively [59].

### Codon usage analysis and genome alignment

CodonW software was used to investigate the distribution of codon usage using the RSCU ratio [60]. To detect divergence hotspots, the online software MAFFT was used to align the whole chloroplast genomes [61]. The whole-genome alignment of *Impatiens* and other species was assessed by the mVISTA program in Shuffle-LAGAN mode [62]. DnaSP was used to calculate nucleotide divergence values using the sliding window method with a window length of 800 bp and a 200 bp step size [63]. Genome-wide alignment with the *H. triflora* chloroplast genome was performed using Mauve software and the MAFFT program [64].

### Phylogenetic analyses

The chloroplast genomes from seven families belonging to the Ericales, including 12 Balsaminaceae species, six Primulaceae species, five Ebenaceae species, four Theaceae species, two Saxifragaceae species, and four Actinidiaceae species were analyzed. Saxifragaceae and Rosaceae species were selected as outgroups due to their closer distances from Ericales as well as ornamental and horticulture value (Supplementary Table S8). The aligned sequences were concatenated with MAFFT version 7.222 and default parameter settings [65]. ML trees were constructed using rapid bootstrapping (1000 replicates) and the search for the best-scoring ML tree option of RAxML v8.2.9 [66]. Based on the Akaike information criterion (AIC) in ModelTest-NG v0.1.6, the best-fitting substitution model (GTR + F + I + G4) was used for ML analyses [67]. The BI tree was generated in MrBayes version 3.2 [68]. The Markov chain Monte Carlo (MCMC) [69] consisted on one million generations with four independent heated chains with sampling after every 1000 generations [70], Burn-in was 10%. The best-fitting substitution model (TVM + F + I) was determined in jModelTest v.2.1.10. The FigTree ver 1.4.2 was used to visualize the output trees [71].

## Supplementary Information

**Additional file 1: Table S1.** Complete chloroplast genomes for 12 Balsaminaceae species. **Table S2.** Distribution of genes for 12 species in Balsaminaceae. **Table S3.** Genes with introns in the chloroplast genomes of newly sequenced Balsaminaceae species. **Table S4.** Codon content of amino acids and stop codons of Balsaminaceae species. **Table S5.** Comparison of long repeats among Balsaminaceae species. **Table S6.** Comparison of SSRs among 12 Balsaminaceae species. **Table S7.** The nucleotide variability (π) values of Balsaminaceae species. **Table S8.** GenBank accession numbers of 40 species used in phylogenetic analysis.

**Additional file 2: Supplementary Figs. S1–6.** Chloroplast genome structure of six *Impatiens* species (*I. chlorosepala, I. fanjingshanica, I. guizhouensis, I. linearisepala, I. loulanensis,* and *I. stenosepala*). **Supplementary Figs. S7–12.** Original pictures of six *Impatiens* species (*I. chlorosepala, I. fanjingshanica, I. guizhouensis, I. linearisepala, I. loulanensis,* and *I. stenosepala*).

## Data Availability

All data generated or analyzed during this study are included in the published article, and the six newly sequenced complete chloroplast genomes were submitted to GenBank under accession numbers MW411293-MW411298. The accession numbers corresponding to the additional datasets used and analyzed in this study can be found in Supplementary Table S8. These data were retrieved from the National Center for Biotechnology Information database.https://www.ncbi.nlm.nih.gov/nuccore/MW411293.1-MW411298.1

## Abbreviations

BI: Bayesian inference
bp: base pairs
ETS: External transcribed spacer
Gb: Gigabases
IGR: Intergenic region
IR: Inverted repeat
ITS: Internal transcribed spacer
LSC: Long single copy
LSR: Long sequence repeat
MCMC: Markov chain Monte Carlo
ML: Maximum likelihood
NCBI: National Center for Biotechnology Information
NGS: Next-generation sequencing
PCR: Polymerase chain reaction
PI: Parsimony informative
rRNA: ribosomal RNA
SSC: Short single copy
SSR: Simple sequence repeat
tRNA: transfer RNA
