# Supplementary material for: Comparative chloroplast genome analysis of Impatiens species (Balsaminaceae) in the karst area of China: insights into genome evolution and phylogenomic implications
Source: BMC Genomics. 2021 Jul 24;22:571. doi: 10.1186/s12864-021-07807-8 (PMC8310579; doi:10.1186/s12864-021-07807-8)
Supplement: Supplementary file 1 — Additional file 1: Table S1. Complete chloroplast genomes for 12 Balsaminaceae species. Table S2. Distribution of genes for 12 species in Balsaminaceae. Table S3. Genes with introns in the chloroplast genomes of newly sequenced Balsaminaceae species. Table S4. Codon content of amino acids and stop codons of Balsaminaceae species. Table S5. Comparison of long repeats among Balsaminaceae species. Table S6. Comparison of SSRs among 12 Balsaminaceae species. Table S7. The nucleotide variability (π) values of Balsaminaceae species. Table S8. GenBank accession numbers of 40 species used in phylogenetic analysis. [file 12864_2021_7807_MOESM1_ESM.zip › Title Page.docx]

Supplementary Information

Comparative chloroplast genome analysis of *Impatiens* species (Balsaminaceae) in the karst area of China: insights into genome evolution and phylogenomic implications

Chao Luo ^1,2^, Wulue Huang ^1^, Huayu Sun ^2^, Huseyin Yer ^2^, Xinyi Li^1^, Yang Li^1^, Bo Yan^1^, Qiong Wang^1^, Yonghui Wen^1^, Meijuan Huang^1^* and Haiquan Huang ^1^*

**Author Details**

1. College of Landscape Architecture and Horticulture Sciences, Southwest Research Center for Engineering Technology of Landscape Architecture(State Forestry and Grassland Administration), Yunnan Engineering Research Center for Functional Flower Resources and Industrialization, Research and Development Center of Landscape Plants and Horticulture Flowers, Southwest Forestry University, Kunming, Yunnan,650224, China.
2. Department of Landscape Architecture and Plant Science, University of Connecticut, Storrs, CT, 06269, USA.

**Supplementary information
Supplementary information**

**Additional file 1:** Table S1: Complete chloroplast genomes for 12 Balsaminaceae species. Table S2: Distribution of genes for 12 species in Balsaminaceae. Table S3: Genes with introns in the chloroplast genomes of newly sequenced Balsaminaceae species. Table S4: Codon content of amino acids and stop codons of Balsaminaceae species. Table S5: Comparison of long repeats among Balsaminaceae species. Table S6: Comparison of SSRs among 12 Balsaminaceae species. Table S7: The nucleotide variability (π) values of Balsaminaceae species. Table S8: GenBank accession numbers of 40 species used in phylogenetic analysis.
