# Supplementary material for: Comparative chloroplast genome analysis of Impatiens species (Balsaminaceae) in the karst area of China: insights into genome evolution and phylogenomic implications
Source: BMC Genomics. 2021 Jul 24;22:571. doi: 10.1186/s12864-021-07807-8 (PMC8310579; doi:10.1186/s12864-021-07807-8)
Supplement: Supplementary file 2 — Additional file 2: Supplementary Figs. S1–6. Chloroplast genome structure of six Impatiens species (I. chlorosepala, I. fanjingshanica, I. guizhouensis, I. linearisepala, I. loulanensis, and I. stenosepala). Supplementary Figs. S7–12. Original pictures of six Impatiens species (I. chlorosepala, I. fanjingshanica, I. guizhouensis, I. linearisepala, I. loulanensis, and I. stenosepala). [file 12864_2021_7807_MOESM2_ESM.zip › Supplementary Figure S1-6.DOCX]

Supplementary Information

Comparative chloroplast genome analysis of *Impatiens* species (Balsaminaceae) in the karst area of China: insights into genome evolution and phylogenomic implications

Chao Luo ^1,2,3, 4^, Wulue Huang ^1,2,3^, Huayu Sun ^4^, Huseyin Yer ^4^, Xinyi Li^1,2,3^, Yang Li^1,2,3^, Bo Yan^1,2,3^, Qiong Wang^1,2,3^, Yonghui Wen^1,2,3^, Meijuan Huang^1,2,3^* and Haiquan Huang ^1,2,3^*

**Author Details**

^1^ College of Landscape Architecture and Horticultural Science, Southwest Forestry University, Kunming, China;

^2^ Research and Development Center of Landscape Plants and Horticulture Flowers, Southwest Forestry University, Kunming, China;

^3^ Engineering Research Center of Functional Flower Resources and Industrialization in Yunnan Province, Kunming, China;

^4^ Department of Landscape Architecture and Plant Science, University of Connecticut, Storrs, CT, 06269, USA;

| **Figure S1 Chloroplast genome structure of *I. chlorosepala*** |
| --- |


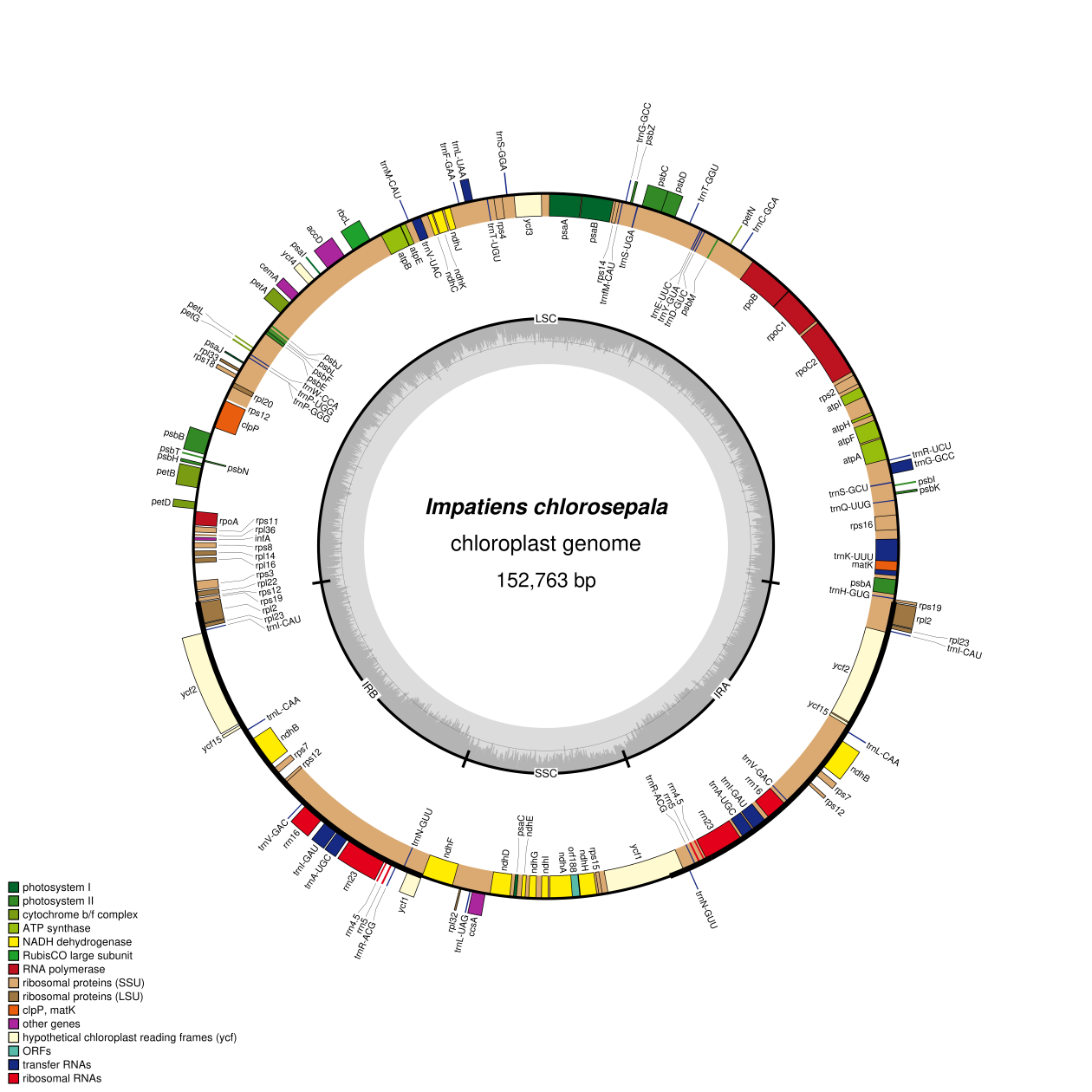


Fig 1. Chloroplast genome structure of Impatiens species. Genes shown outside the map circles are transcribed clockwise, while those drawn inside are transcribed counterclockwise. Genes from different functional groups are color-coded according to the key at the top right. The positions of long single copy (LSC), short single copy (SSC), and two inverted repeats (IR: IRA and IRB) regions are shown in the inner circles.

| **Figure S2 Chloroplast genome structure of *I. fanjingshanica*** |
| --- |


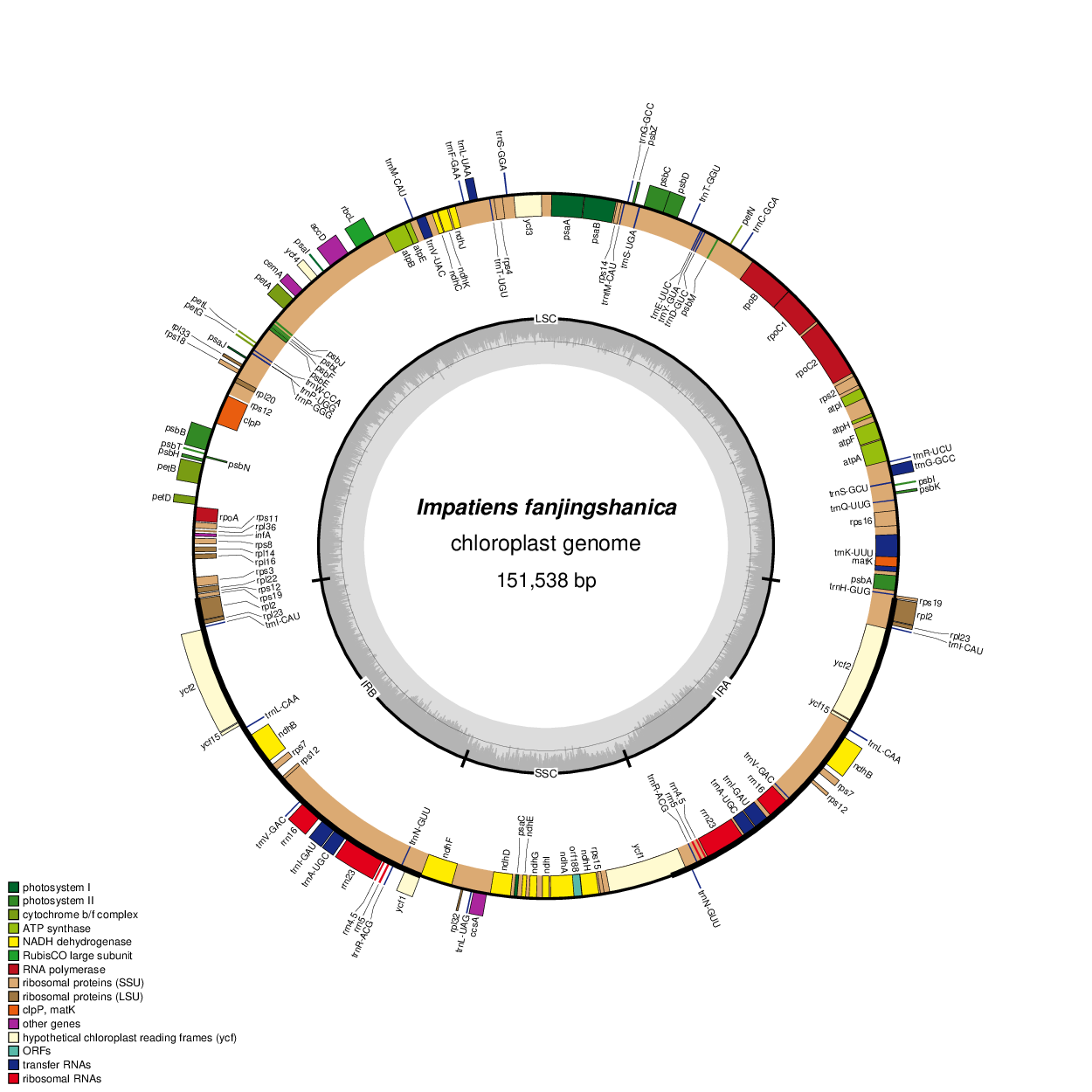


Fig 2. Chloroplast genome structure of Impatiens species. Genes shown outside the map circles are transcribed clockwise, while those drawn inside are transcribed counterclockwise. Genes from different functional groups are color-coded according to the key at the top right. The positions of long single copy (LSC), short single copy (SSC), and two inverted repeats (IR: IRA and IRB) regions are shown in the inner circles.

| **Figure S3 Chloroplast genome structure of *I. guizhouensis*** |
| --- |


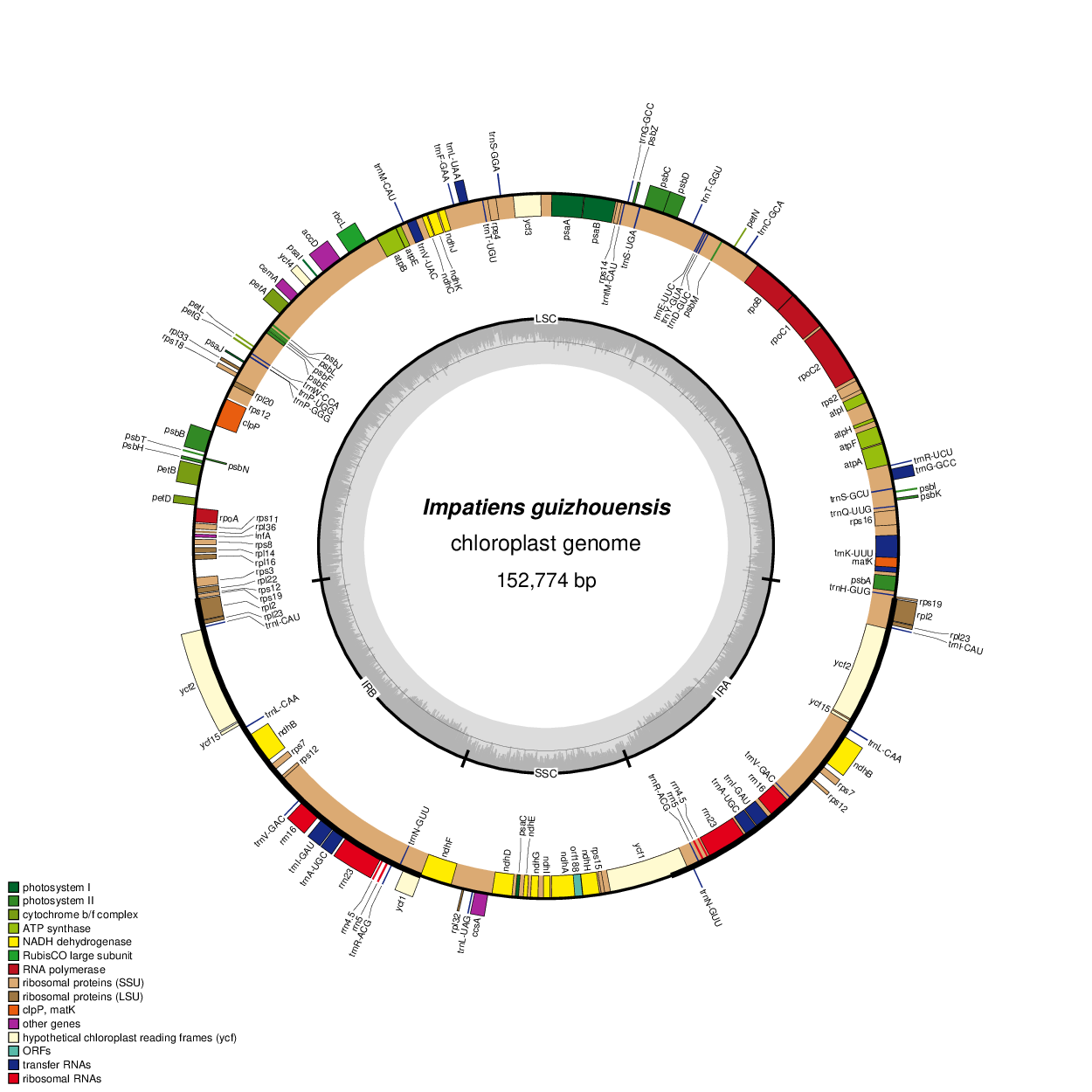


Fig 3. Chloroplast genome structure of Impatiens species. Genes shown outside the map circles are transcribed clockwise, while those drawn inside are transcribed counterclockwise. Genes from different functional groups are color-coded according to the key at the top right. The positions of long single copy (LSC), short single copy (SSC), and two inverted repeats (IR: IRA and IRB) regions are shown in the inner circles.

| **Figure S4 Chloroplast genome structure of *I. linearisepala*** |
| --- |


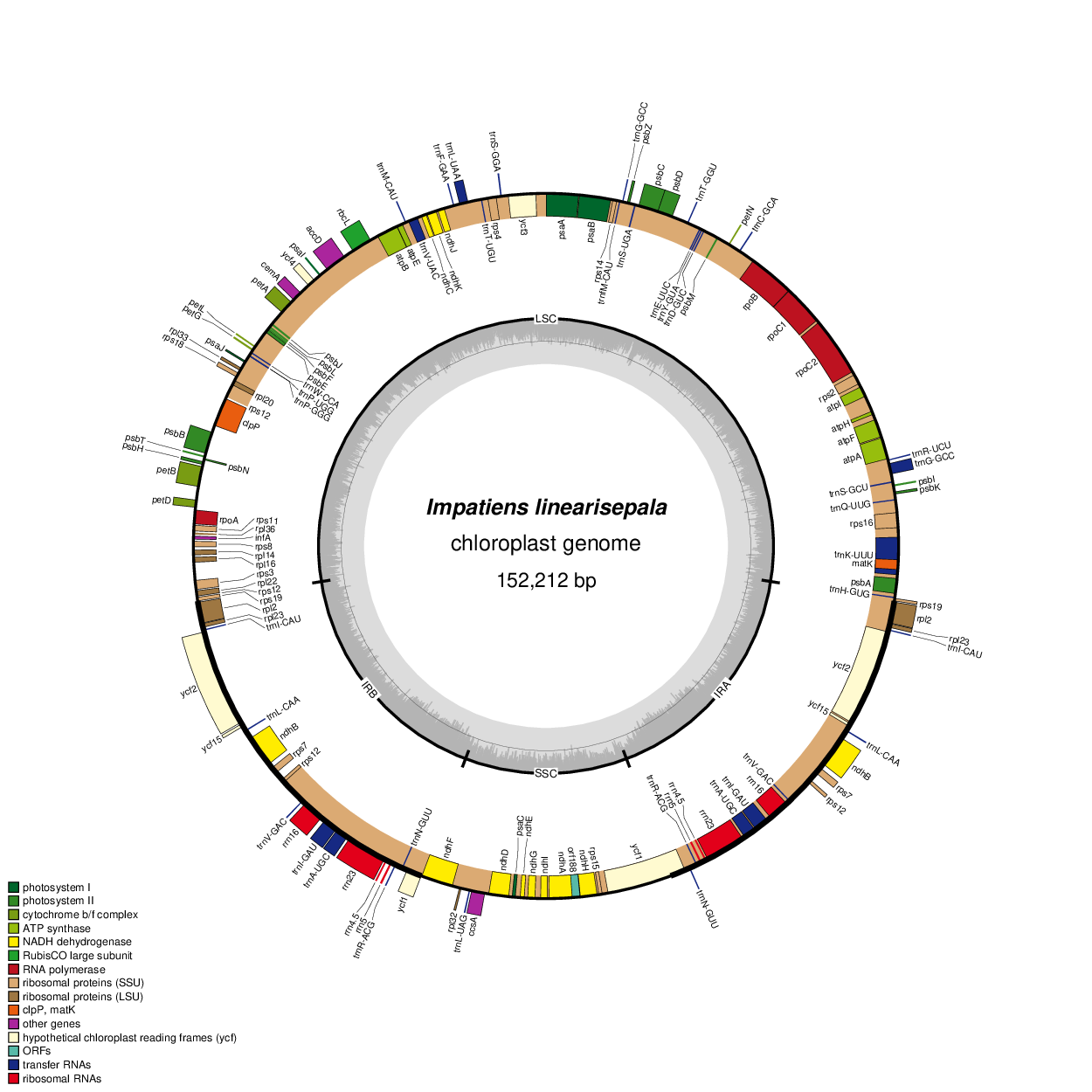


Fig 4. Chloroplast genome structure of Impatiens species. Genes shown outside the map circles are transcribed clockwise, while those drawn inside are transcribed counterclockwise. Genes from different functional groups are color-coded according to the key at the top right. The positions of long single copy (LSC), short single copy (SSC), and two inverted repeats (IR: IRA and IRB) regions are shown in the inner circles.

| **Figure S5 Chloroplast genome structure of *I. loulanensis*** |
| --- |


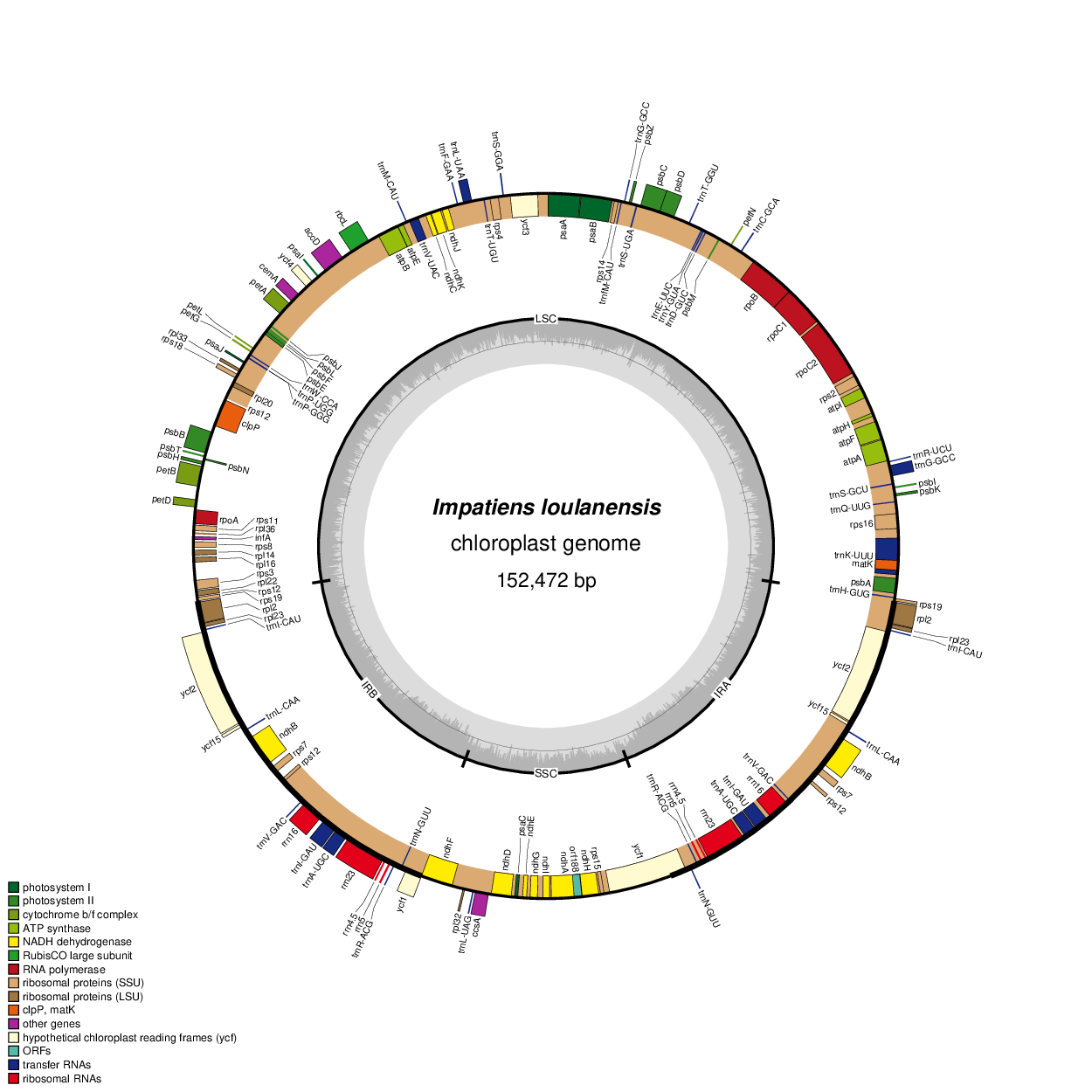


Fig 5. Chloroplast genome structure of Impatiens species. Genes shown outside the map circles are transcribed clockwise, while those drawn inside are transcribed counterclockwise. Genes from different functional groups are color-coded according to the key at the top right. The positions of long single copy (LSC), short single copy (SSC), and two inverted repeats (IR: IRA and IRB) regions are shown in the inner circles.

| **Figure S6 Chloroplast genome structure of *I. stenosepala*** |
| --- |


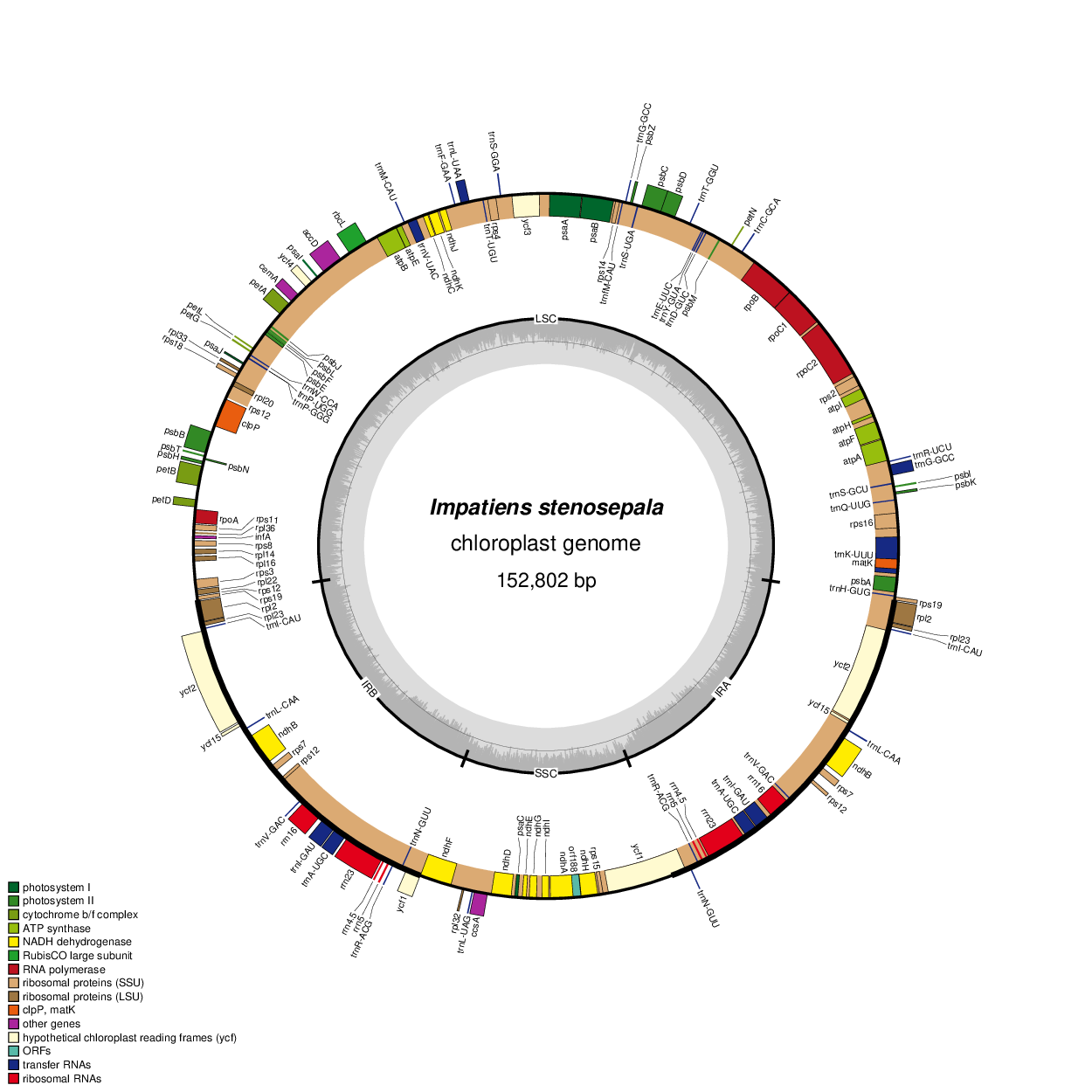


Fig 6. Chloroplast genome structure of Impatiens species. Genes shown outside the map circles are transcribed clockwise, while those drawn inside are transcribed counterclockwise. Genes from different functional groups are color-coded according to the key at the top right. The positions of long single copy (LSC), short single copy (SSC), and two inverted repeats (IR: IRA and IRB) regions are shown in the inner circles.
