# Supplementary material for: Comparative chloroplast genome analysis of Impatiens species (Balsaminaceae) in the karst area of China: insights into genome evolution and phylogenomic implications
Source: BMC Genomics. 2021 Jul 24;22:571. doi: 10.1186/s12864-021-07807-8 (PMC8310579; doi:10.1186/s12864-021-07807-8)

Supplementary Information

Comparative chloroplast genome analysis of Impatiens species (Balsaminaceae) in the karst area of China: insights into genome evolution and phylogenomic implications

Chao Luo [1,2], Wulue Huang [1], Huayu Sun [2], Huseyin Yer [2], Xinyi Li[1], Yang Li[1], Bo Yan[1], Qiong Wang[1], Yonghui Wen[1], Meijuan Huang[1]* and Haiquan Huang [1]*

Author Details

1 College of Landscape Architecture and Horticulture Sciences, Southwest Research Center for Engineering Technology of Landscape Architecture(State Forestry and Grassland Administration), Yunnan Engineering Research Center for Functional Flower Resources and Industrialization,Research and Development Center of Landscape Plants and Horticulture Flowers, Southwest Forestry University, Kunming, Yunnan,650224, China.

2 Department of Landscape Architecture and Plant Science, University of Connecticut, Storrs, CT, 06269, USA.

Figure S7 Orginnal picture of *I. chlorosepala*

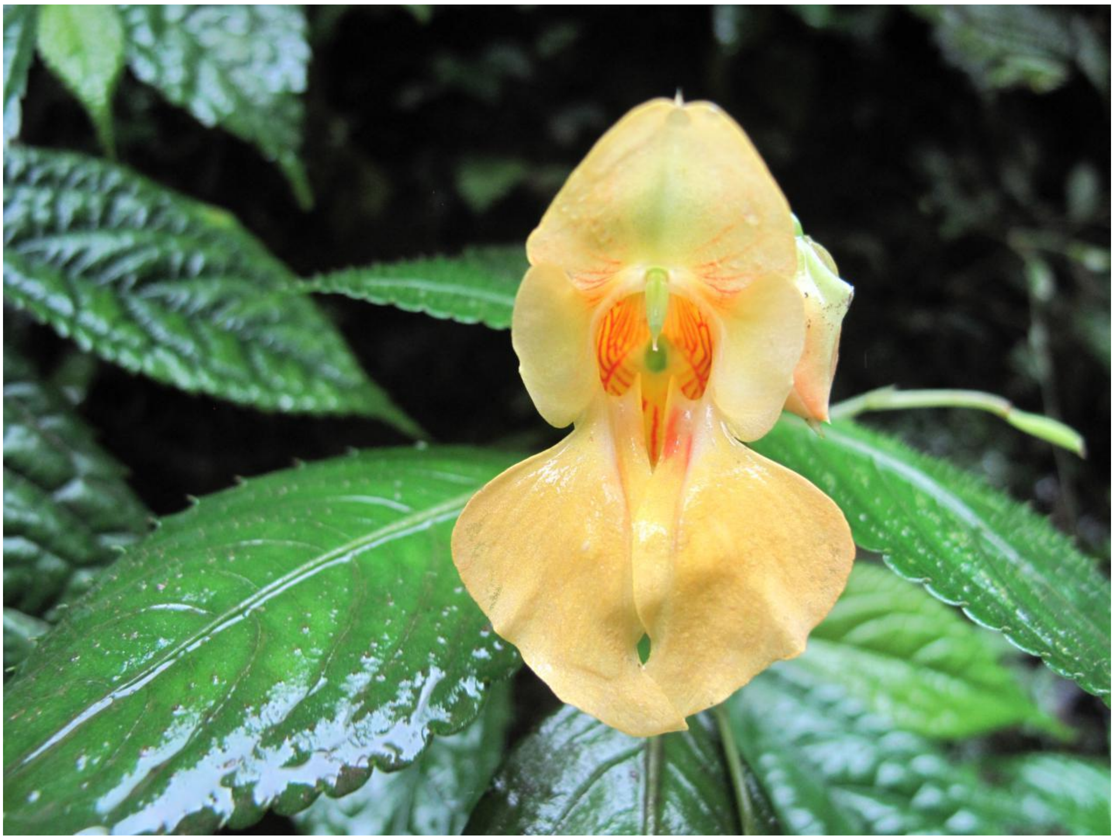

Figure S8 Orginnal picture of *I. fanjingshanica*

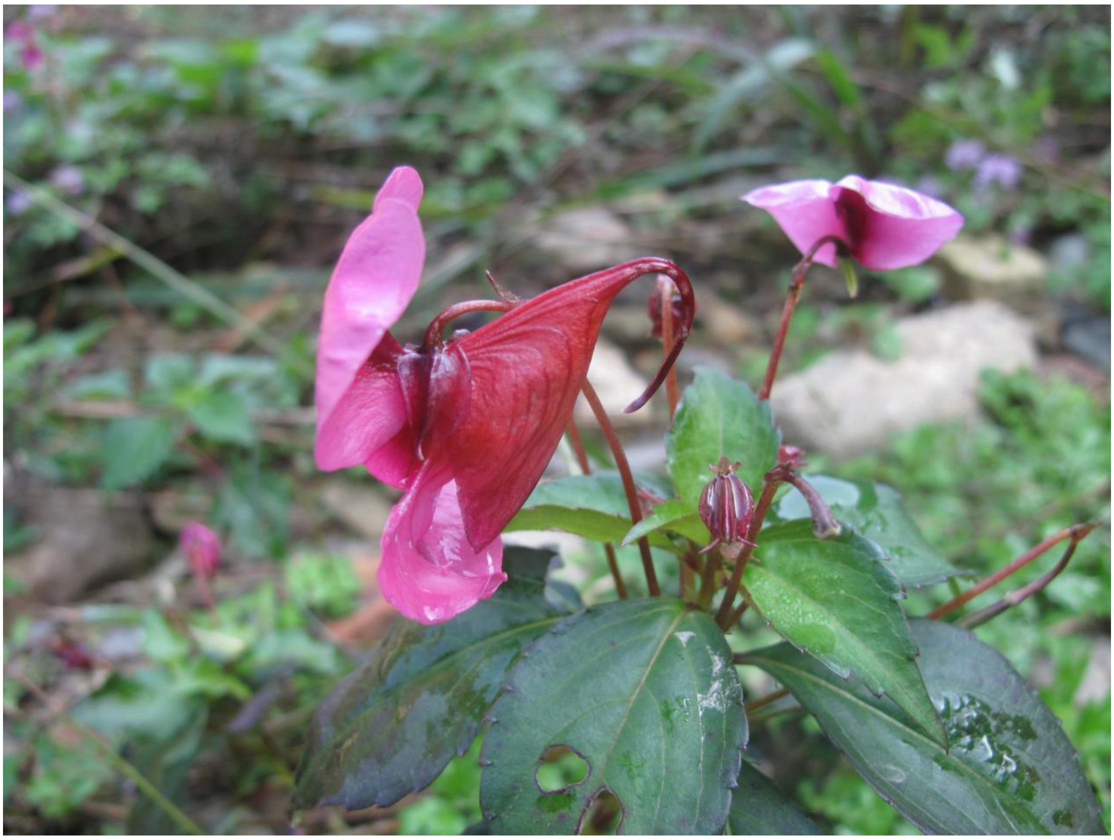

Figure S9 Orginnal picture of *I. guizhouensis*

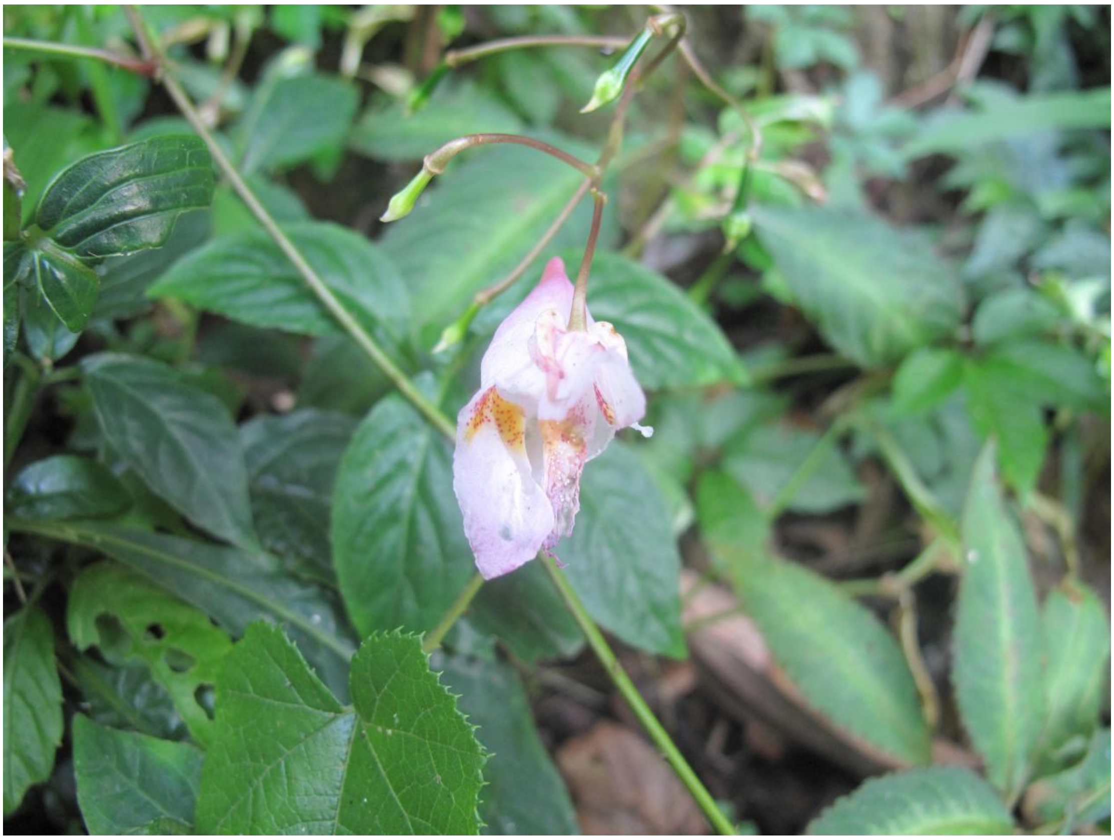

Figure S10 Orginnal picture of *I. linearisepala*

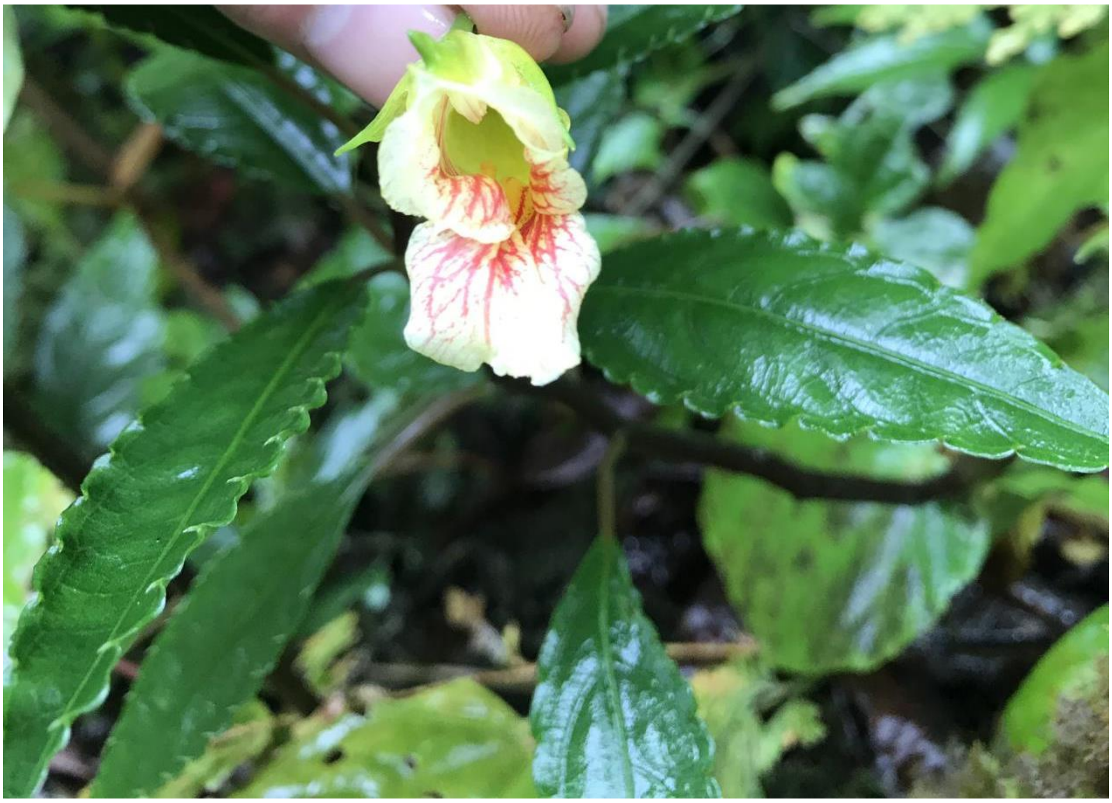

Figure S11 Orginnal picture of *I. loulanensis*

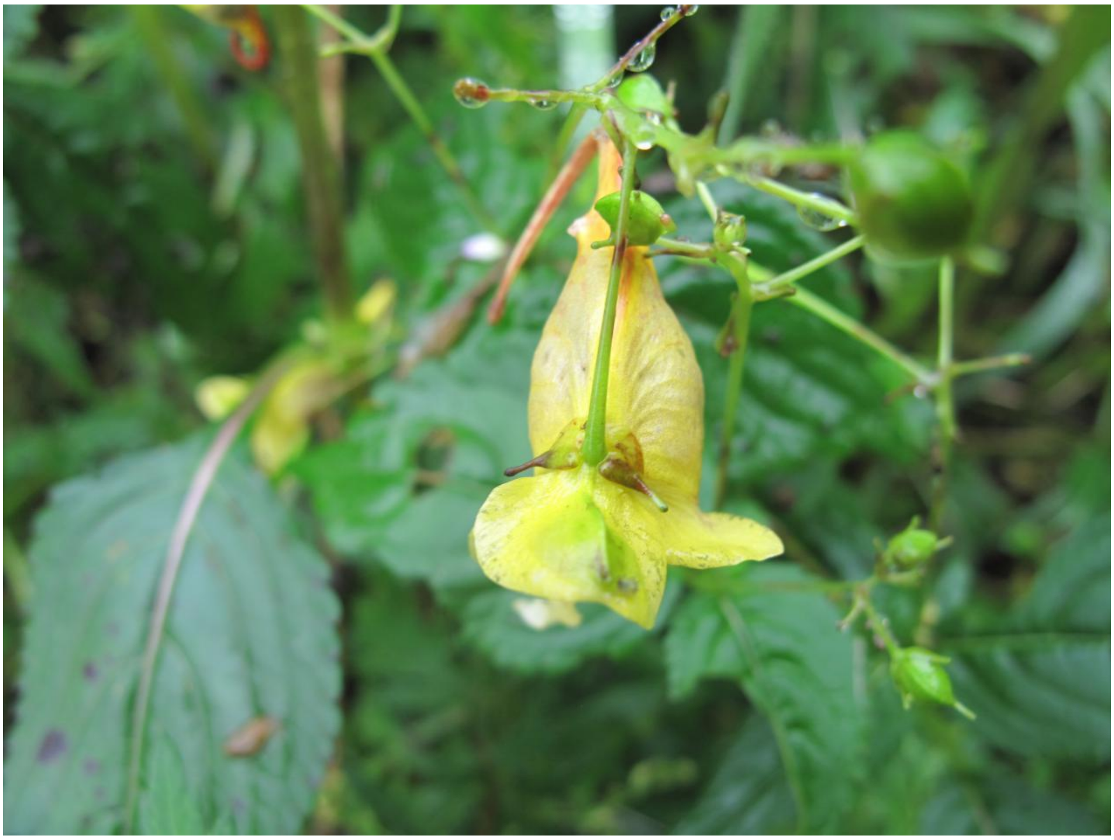

Figure S12 Orginnal picture of *I. stenosepala*

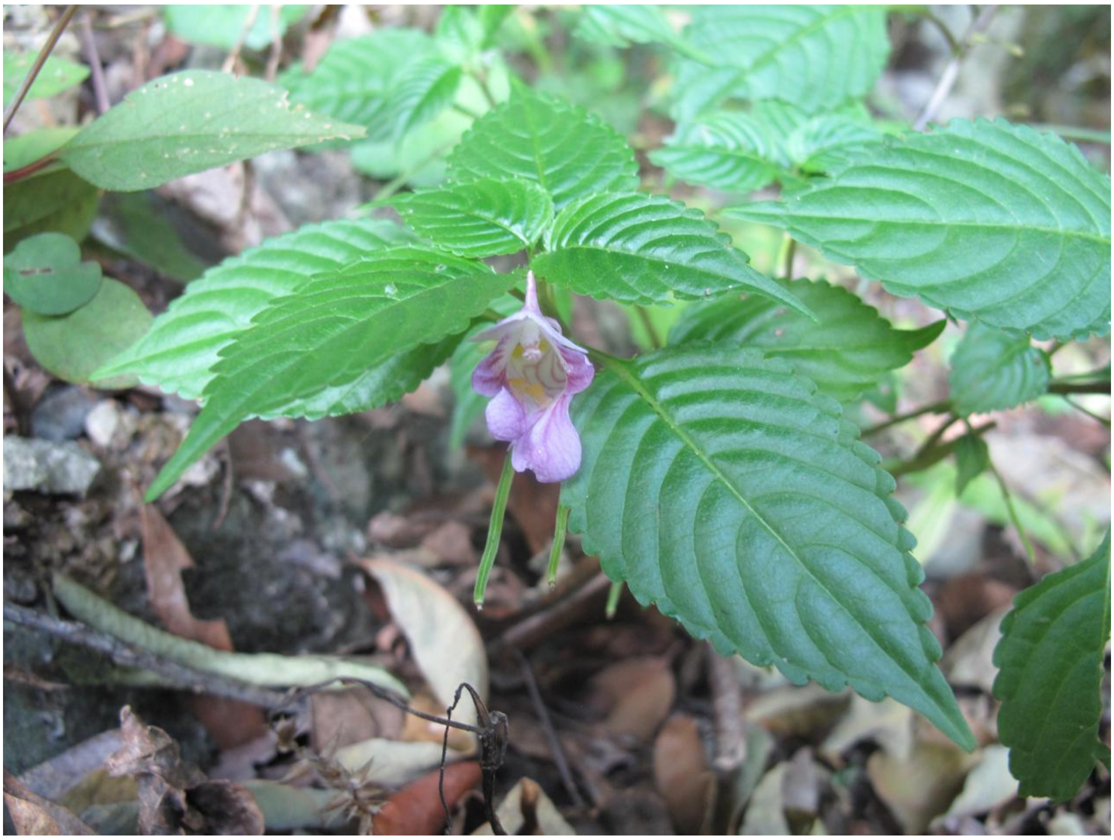

Supplement: Supplementary file 2 — Additional file 2: Supplementary Figs. S1–6. Chloroplast genome structure of six Impatiens species (I. chlorosepala, I. fanjingshanica, I. guizhouensis, I. linearisepala, I. loulanensis, and I. stenosepala). Supplementary Figs. S7–12. Original pictures of six Impatiens species (I. chlorosepala, I. fanjingshanica, I. guizhouensis, I. linearisepala, I. loulanensis, and I. stenosepala). [file 12864_2021_7807_MOESM2_ESM.zip › Supplementary Figure S7-12.pdf]
